# Crystal structures of [Cu(phen)(H_2_O)_3_(*M*F_6_)]·H_2_O (*M* = Ti, Zr, Hf) and [Cu(phen)(H_2_O)_2_F]_2_[HfF_6_]·H_2_O

**DOI:** 10.1107/S2056989021000645

**Published:** 2021-01-26

**Authors:** Matthew L. Nisbet, Kenneth R. Poeppelmeier

**Affiliations:** a 2145 Sheridan Road, Evanston, IL 60208, USA

**Keywords:** crystal structures, lambda (Λ)-shapes, *d*^0^ early transition metals, hydro­thermal synthesis

## Abstract

The crystal structures of three bridged bimetallic mol­ecular compounds, namely, tri­aqua-2κ^3^ *O*-μ-fluorido-penta­fluorido-1κ^5^ *F*-(1,10-phenanthroline-2κ^2^ *N*,*N*′)copper(II)titanium(IV) monohydrate, [Cu(TiF_6_)(phen)(H_2_O)_3_]·H_2_O (phen is 1,10-phenanthroline, C_12_H_8_N_2_), (I), tri­aqua-2κ^3^ *O*-μ-fluorido-penta­fluorido-1κ^5^ *F*-(1,10-phenanthroline-2κ^2^ *N*,*N*′)copper(II)zirconium(IV) monohydrate, [Cu(ZrF_6_)(phen)(H_2_O)_3_]·H_2_O, (II), and tri­aqua-2κ^3^ *O*-μ-fluorido-penta­fluorido-1κ^5^ *F*-(1,10-phenanthroline-2κ^2^ *N*,*N*′)copper(II)hafnium(IV) monohydrate, [Cu(HfF_6_)(phen)(H_2_O)_3_]·H_2_O, (III), and one mol­ecular salt, bis­[diaqua­fluorido­(1,10-phenanthroline-κ^2^ *N*,*N*′)copper(II)] hexa­fluorido­hafnate(IV) dihydrate, [CuF(phen)(H_2_O)_2_]_2_[HfF_6_]·2H_2_O, (IV), are reported. The bridged bimetallic compounds adopt Λ-shaped configurations, with the octa­hedrally coordinated copper(II) center linked to the fluorinated early transition metal *via* a fluoride linkage. The extended structures of these Λ-shaped compounds are organized through both intra- and inter­molecular hydrogen bonds and inter­molecular π–π stacking. The salt compound [Cu(phen)(H_2_O)_2_F]_2_[HfF_6_]·H_2_O displays an isolated square-pyramidal Cu(phen)(H_2_O)_2_F^+^ complex linked to other cationic complexes and isolated HfF_6_^2−^ anions through inter­molecular hydrogen-bonding inter­actions.

## Abstract 2

Crystal structures of three compounds with the formula [Cu(phen)(H_2_O)_3_(*M*F_6_)]·H_2_O (*M* = Ti, Zr, Hf) based on bimetallic Λ-shaped mol­ecules and a related salt with the formula [Cu(phen)(H_2_O)_2_F]_2_[HfF_6_]·H_2_O are reported.

## Chemical context   

Lambda (Λ)-shaped mol­ecules have been demonstrated as efficient building blocks in the synthesis of non-centrosymmetric (NCS) materials *via* arrangement into head-to-tail and accordion (head-to-head, tail-to-tail) structures (Yamamoto *et al.*, 1992 ▸; Tao *et al.*, 1994 ▸, 1995 ▸; Ostroverkhov *et al.*, 2001 ▸; Chang *et al.*, 2009 ▸). Although this concept was first applied to organic Λ-shaped mol­ecules in crystalline materials and polymers, recently NCS compounds based on inorganic bimetallic Λ-shapes have been reported, namely K_10_(Mo_2_O_4_F_7_)_3_ *X* (*X* = Cl, ([Br_3_][Br])_1/2_, ([I_3_][I])_1/2_), K_10_(Nb_2_O_2_F_9_)_3_X (X = Br, ([Br_3_][Br])_1/2_, ([I_3_][I])_1/2_), and [Cu(H_2_O)_5_(VOF_4_(H_2_O))]·H_2_O (Donakowski *et al.*, 2012 ▸; Holland *et al.*, 2014 ▸). Here, we report the structures of three centrosymmetric compounds based on inorganic bimetallic Λ-shapes with the formula [Cu(phen)(H_2_O)_3_(*M*F_6_)]·H_2_O (*M* = Ti, Zr, Hf; phen = 1,10-phenanthroline). Although these compounds crystallize with inversion symmetry, the novel mol­ecular building units are potential targets of future studies aimed to perturb their packing arrangement to form NCS structures. The salt compound [Cu(phen)(H_2_O)_2_F]_2_[HfF_6_]·H_2_O provides a point of comparison as an unbridged analogue of [Cu(phen)(H_2_O)_3_(HfF_6_)]·H_2_O.
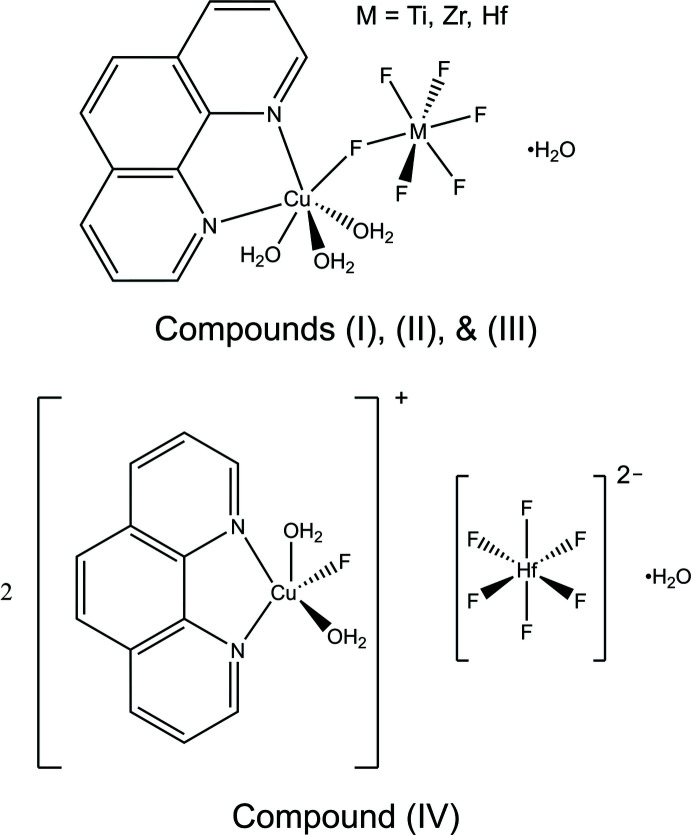


## Structural commentary   

**Figure 1:**
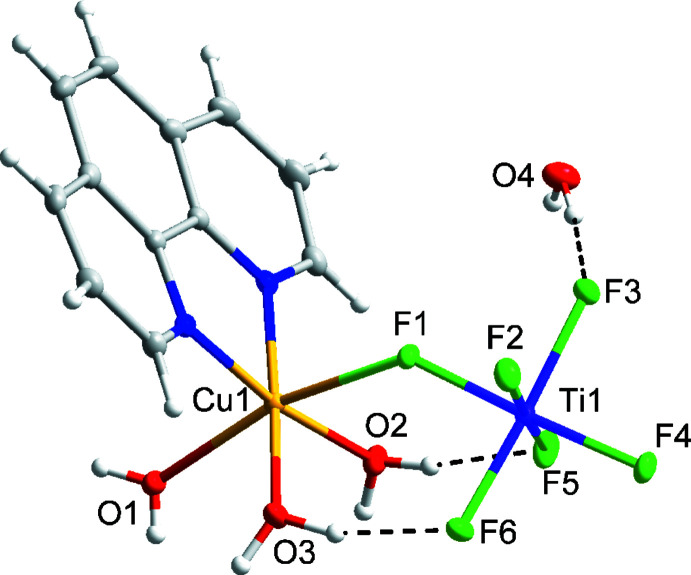
Mol­ecular structure of compound (I), [Cu(phen)(H_2_O)_3_(TiF_6_)]·H_2_O. Ellipsoids of non-H atoms are drawn at 50% probability. H atoms are drawn with an atomic radius of 0.135 Å.

Compound (I) has the formula [Cu(phen)(H_2_O)_3_(TiF_6_)]·H_2_O and crystallizes in the ortho­rhom­bic space group *Pbca* (Fig. 1 ▸). The structure of compound (I) features Cu1 in a tetra­gonally distorted octa­hedral environment with elongated axial Cu1—F1 [2.3643 (12) Å] and Cu1—O1 [2.2794 (17) Å] bonds owing to the Jahn–Teller effect of copper(II). The Cu1 center is linked to the TiF_6_^2−^ anion through the bridging F1 ligand. The octa­hedral coordination environment of Ti1 is slightly distorted, with Ti1—F bond lengths ranging from 1.8395 (13) to 1.9035 (13) Å. The Λ-shape, indicated by the Cu1—F1—Ti1 bond angle of 134.93 (6)°, is enforced by the two intra­molecular O2—H2*B*⋯F5 and O3—H3*B*⋯F6 hydrogen bonds (Table 1 ▸).

**Figure 2:**
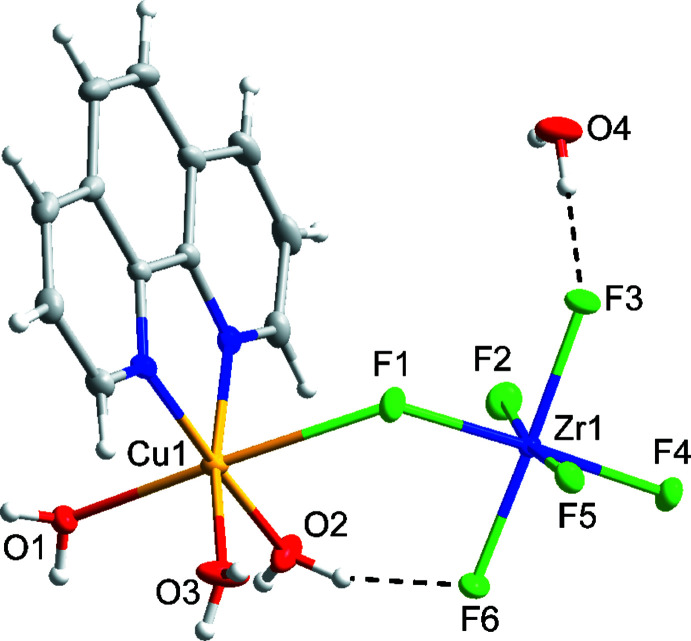
Mol­ecular structure of compound (II), [Cu(phen)(H_2_O)_3_(ZrF_6_)]·H_2_O. Ellipsoids of non-H atoms are drawn at 50% probability. H atoms are drawn with an atomic radius of 0.135 Å.

Compound (II) has the formula [Cu(phen)(H_2_O)_3_(ZrF_6_)]·H_2_O and crystallizes in the monoclinic space group *P*2_1_ */n* (Fig. 2 ▸). The structure of compound (II) features Cu1 in a tetra­gonally distorted octa­hedral environment with elongated axial Cu1—F1 [2.5184 (6) Å] and Cu—O1 [2.2758 (7) Å] bonds owing to the Jahn–Teller effect of copper(II). The Cu1 center is linked to the ZrF_6_^2−^ anion through the bridging F1 ligand. The octa­hedral coordination environment of Zr1 is slightly distorted, with Zr1—F bond lengths ranging from 1.9910 (6) to 2.0430 (6) Å. The Λ-shape, indicated by the Cu1—F1—Zr1 bond angle of 132.59 (3)°, is enforced by an intra­molecular O2—H2*B*⋯F6 hydrogen bond (Table 2 ▸). The single intra­molecular hydrogen bond in compound (II) tilts the ZrF_6_^2−^ group significantly relative to the TiF_6_^2−^ group in compound (I), which is depicted in Fig. 5 ▸ and reflected in the F1—Cu1—N1 bond angle of 77.75 (3)° angle in compound (II) compared to 89.45 (6)° in compound (I).

**Figure 3:**
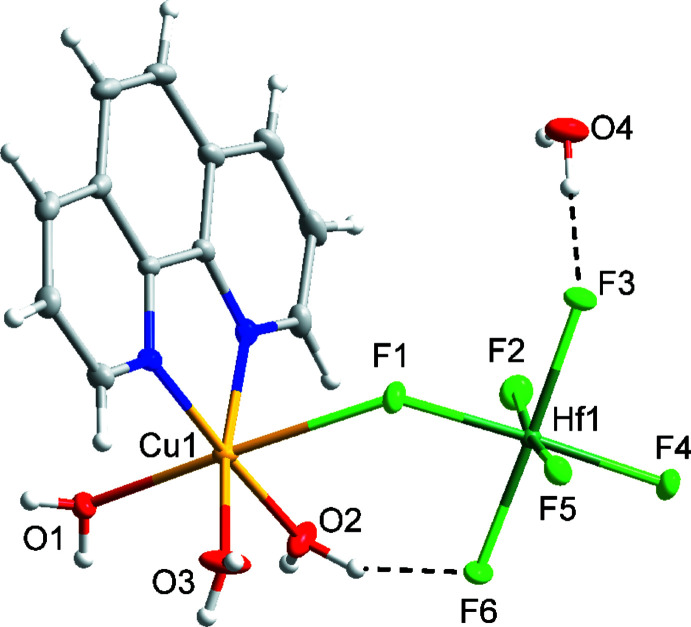
Mol­ecular structure of compound (III), [Cu(phen)(H_2_O)_3_(HfF_6_)]·H_2_O. Ellipsoids of non-H atoms are drawn at 50% probability. H atoms are drawn with an atomic radius of 0.135 Å.

Compound (III) has the formula [Cu(phen)(H_2_O)_3_(HfF_6_)]·H_2_O and crystallizes in the monoclinic space group *P*2_1_ */n* (Fig. 3 ▸). Compound (III) is isostructural to compound (II).

**Figure 4:**
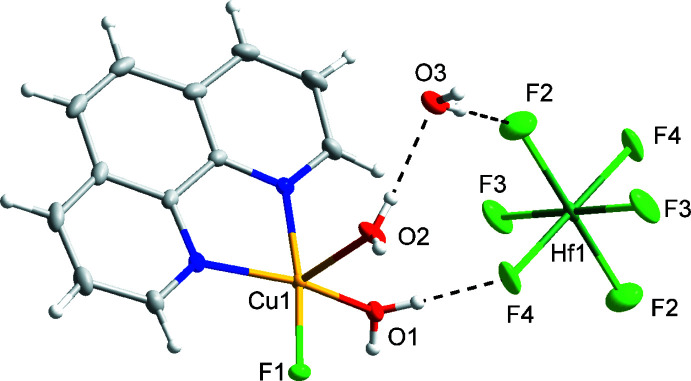
Mol­ecular structure of compound (IV), [Cu(phen)(H_2_O)_2_F]_2_[HfF_6_]·H_2_O. Ellipsoids of non-H atoms are drawn at 50% probability. H atoms are drawn with an atomic radius of 0.135 Å.

**Figure 5:**
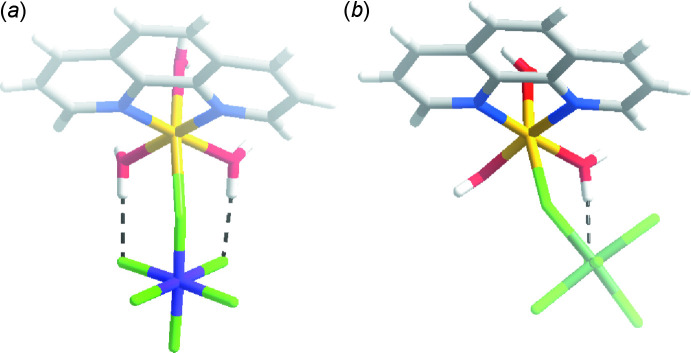
Comparison of the mol­ecular structures of (*a*) compound (I) and (*b*) compound (III).

Compound (IV) has the formula [Cu(phen)(H_2_O)_2_F]_2_[HfF_6_]·H_2_O and crystallizes in the monoclinic space group *P*2_1_ */n* (Fig. 4 ▸). The structure of compound (IV) features isolated square pyramidal Cu(phen)(H_2_O)_2_F^+^ cations and octa­hedral HfF_6_^2−^ anions. The free HfF_6_^2−^ octa­hedron occupies an inversion center with three distinct bond lengths ranging between 1.9863 (10) and 1.9957 (9) Å.

## Supra­molecular features   

The Λ-shaped building units in compounds (I)–(III) are arranged in head-to-tail chains via inter­molecular hydrogen bonding with multiple hydrogen-bonding inter­actions and π–π stacking contacts to adjacent chains.

**Figure 6:**
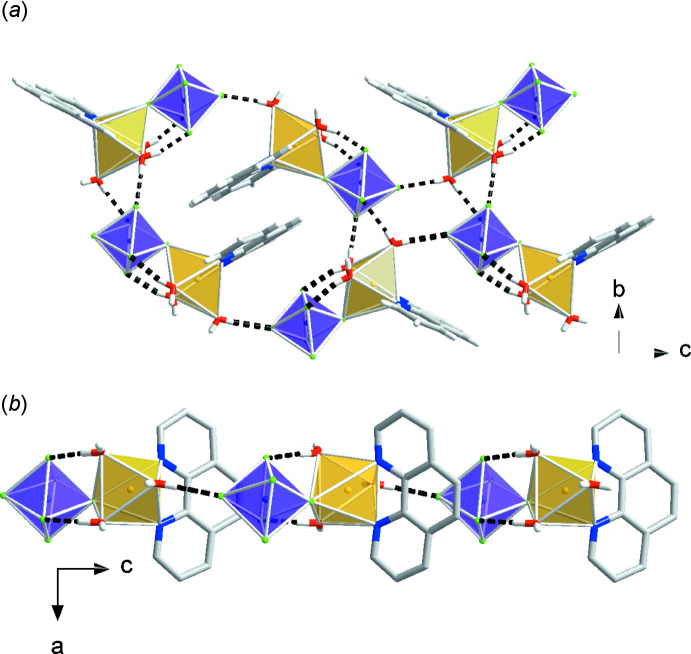
Packing diagrams of compound (I), [Cu(phen)(H_2_O)_3_(TiF_6_)]·H_2_O. Yellow polyhedra represent Cu(phen)(H_2_O)_3_^2+^ cations and purple polyhedra represent TiF_6_^2−^ anions.

Each [Cu(phen)(H_2_O)_3_(TiF_6_)] complex in compound (I) participates in hydrogen bonding with four other [Cu(phen)(H_2_O)_3_(TiF_6_)] complexes and three free water mol­ecules (Fig. 6 ▸, Table 1 ▸). The complexes pack with both face-to-face and displaced π–π stacking inter­actions (Table 5 ▸).

**Figure 7:**
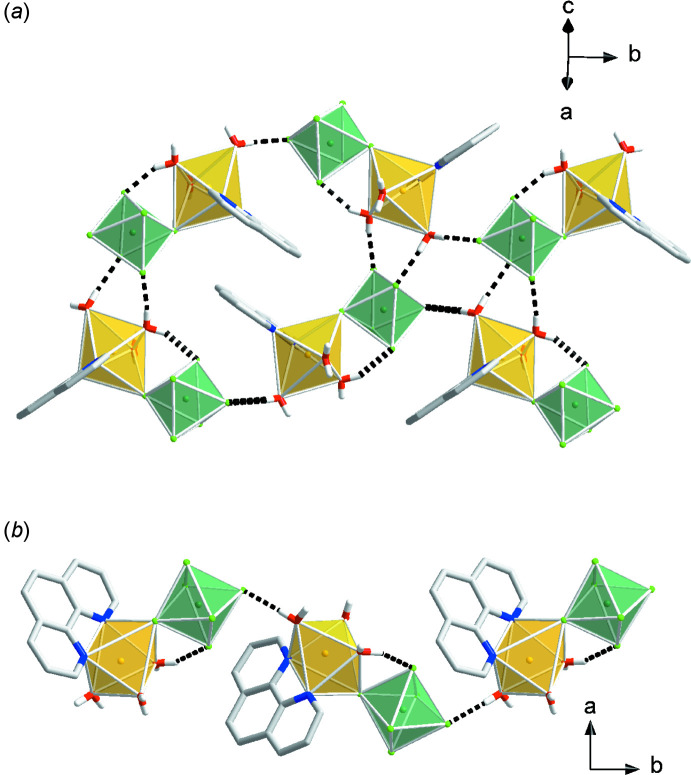
Packing diagrams of compound (II), [Cu(phen)(H_2_O)_3_(ZrF_6_)]·H_2_O, and compound (III), [Cu(phen)(H_2_O)_3_(HfF_6_)]·H_2_O. Yellow polyhedra represent Cu(phen)(H_2_O)_3_^2+^ cations and green polyhedra represent ZrF_6_^2−^ or HfF_6_^2−^ anions.

The [Cu(phen)(H_2_O)_3_(*M*F_6_)] (*M* = Zr, Hf) units in compound (II) and compound (III) are involved in five hydrogen-bonding contacts to adjacent [Cu(phen)(H_2_O)_3_(*M*F_6_)] complexes and three contacts to hydrating water mol­ecules (Fig. 7 ▸, Table 2 ▸, and Table 3 ▸). The [Cu(phen)(H_2_O)_3_(*M*F_6_)] complexes participate in parallel displaced π–π stacking inter­actions (Table 5 ▸).

**Figure 8:**
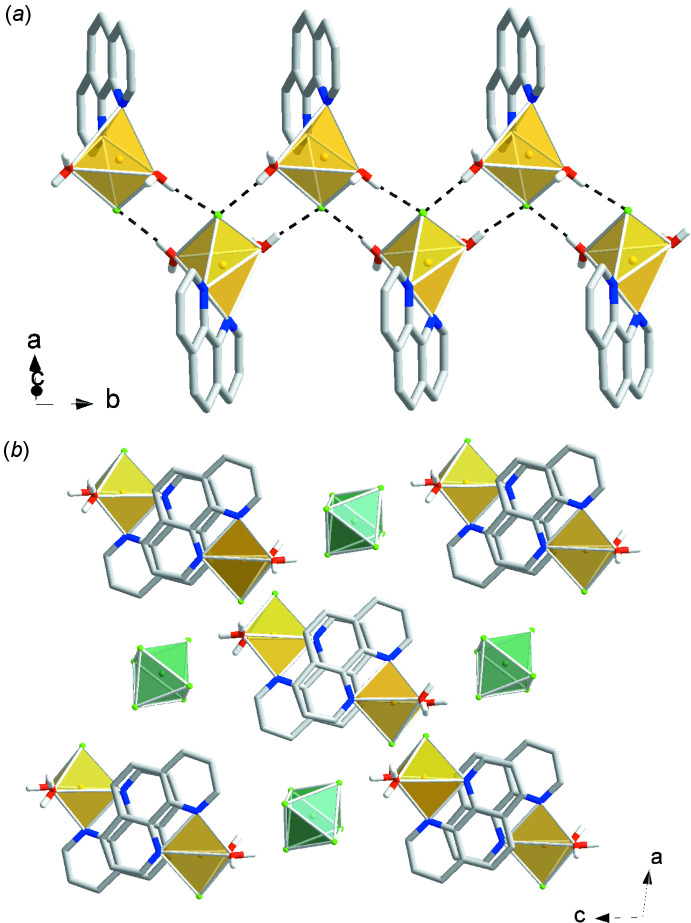
Packing diagrams of compound (IV), [Cu(phen)(H_2_O)_2_F]_2_[HfF_6_]·H_2_O. Yellow polyhedra represent Cu(phen)(H_2_O)_2_F^+^ cations and green polyhedra represent HfF_6_^2−^ anions.

In compound (IV), each fluoride ligand forms two hydrogen bonds with the water ligands of adjacent Cu(phen)(H_2_O)_2_F^+^ complexes (Fig. 8 ▸). The equatorial water ligands form O1—H1*A*⋯F1 hydrogen bonds with adjacent Cu(phen)(H_2_O)_2_F^+^ complexes and O1—H1*B*⋯F4 hydrogen bonds with HfF_6_^2−^ groups (Table 4 ▸). The apical water mol­ecule forms an O2—H2*B*⋯F1 hydrogen bond to an adjacent Cu(phen)(H_2_O)_2_F^+^ complex and a O2—H2*A*⋯O3 hydrogen bond with a free water mol­ecule (Table 4 ▸). Each *M*F_6_^2−^ group forms hydrogen bonds with four free water mol­ecules and two Cu(phen)(H_2_O)_2_F^+^ complexes. The Cu(phen)(H_2_O)_2_F^+^ complexes pack with both face-to-face and parallel displaced π–π stacking inter­actions (Table 5 ▸).

## Database survey   

Aside from compounds (I), (II), and (III), the compound [Cu(H_2_O)_5_(VO(H_2_O)F_4_)]·H_2_O (Donakowski *et al.*, 2012 ▸) is the only example of a mol­ecular inorganic Λ-shape known to the authors. [Cu(H_2_O)_5_(VOF_4_(H_2_O))]·H_2_O contains a mol­ecular Λ-shaped [Cu(H_2_O)_5_(VOF_4_(H_2_O))] mol­ecule that is bridged *via* the Cu1—O8—V1 linkage with a bond angle of 142.88°. The Λ-shape of this complex is supported by a single intra­molecular hydrogen bond as well as two hydrogen-bonding inter­actions with a free water mol­ecule that serves as an inter­molecular ‘bridging mol­ecule’. In contrast, the hydrating water mol­ecules in compounds (I), (II), and (III) bridge between adjacent complexes rather than the same complex. The smallest O8—Cu—O bond angle in [Cu(H_2_O)_5_(VOF_4_(H_2_O))]·H_2_O is 88.42°, meaning that the complex has a small tilt similar to compound (I).

The Λ-shapes in [Cu(H_2_O)_5_(VO(H_2_O)F_4_)]·H_2_O are arranged in a polar NCS lattice containing head-to-head/tail-to-tail chains in which the polar moments of the Λ-shaped complexes are partially aligned perpendicular to the chain direction, with head-to-tail orientations between chains. In contrast, the Λ-shapes found in compounds (I), (II), and (III) are arranged in non-polar head-to-tail chains in which the polar moments of the Λ-shaped complexes are arranged in an anti­parallel fashion within the chain, with a head-to-tail arrangement between chains.

## Synthesis and crystallization   

The compounds reported here were synthesized by the hydro­thermal pouch method (Harrison *et al.*, 1993 ▸). In each reaction, reagents were heat-sealed in Teflon pouches. Groups of six pouches were then placed into a 125 mL Parr autoclave with 40 mL of distilled water as backfill. The autoclave was heated at a rate of 5 K min^−1^ to 423 K and held at 423 K for 24 h. The autoclaves were allowed to cool to room temperature at a rate of 6 K h^−1^. Solid products were recovered by vacuum filtration. Compound (I) was synthesized in a pouch containing 1.69 mmol of CuO, 1.69 mmol of TiO_2_, 2.56 mmol of 1,10-phenanthroline, 1.0 mL (27.6 mmol) of HF(*aq*) (48%), and 0.1 mL (5.5 mmol) of deionized H_2_O. Compound (II) was synthesized in a pouch containing 1.69 mmol of CuO, 1.69 mmol of ZrO_2_, 2.56 mmol of phen, 1.0 mL (27.6 mmol) of HF(*aq*) (48%), and 0.1 mL (5.5 mmol) of deionized H_2_O. Compound (III) was synthesized in a pouch containing 1.69 mmol of CuO, 1.69 mmol of HfO_2_, 2.56 mmol of phen, 1.0 mL (27.6 mmol) of HF(*aq*) (48%), and 0.1 mL (5.5 mmol) of deionized H_2_O. Compound (IV) was synthesized in a pouch containing 1.69 mmol of CuO, 1.69 mmol of HfO_2_, 2.56 mmol of phen, 0.4 mL (11.03 mmol) of HF(*aq*) (48%), and 0.7 mL (38.85 mmol) of deionized H_2_O.

## Refinement   

Crystal data, data collection and structure refinement details are summarized in Table 6 ▸. Non-hydrogen atoms were refined with anisotropic displacement parameters. Hydrogen-atom positions were assigned from difference map peaks with the exception of C—H hydrogen atoms of 1,10-phenanthroline, which were constrained to ride at distances of 0.95 Å from the associated C atoms with *U*_iso_(H) = 1.2*U*_eq_(C) within *OLEX2* (Dolomanov *et al.*, 2009).

## Supplementary Material

Crystal structure: contains datablock(s) I, II, III, IV, I, II, III, IV. DOI: 10.1107/S2056989021000645/tx2034sup1.cif

Structure factors: contains datablock(s) I. DOI: 10.1107/S2056989021000645/tx2034Isup2.hkl

Structure factors: contains datablock(s) II. DOI: 10.1107/S2056989021000645/tx2034IIsup3.hkl

Structure factors: contains datablock(s) III. DOI: 10.1107/S2056989021000645/tx2034IIIsup4.hkl

Structure factors: contains datablock(s) IV. DOI: 10.1107/S2056989021000645/tx2034IVsup5.hkl

CCDC references: 2045776, 2045775, 2045774, 2045773

Additional supporting information:  crystallographic information; 3D view; checkCIF report

## Figures and Tables

**Table 1:** Hydrogen-bond geometry (Å, °) for (I)

*D*—H⋯*A*	*D*—H	H⋯*A*	*D*⋯*A*	*D*—H⋯*A*
O1—H1*A*⋯F2^i^	0.70 (4)	2.08 (4)	2.775 (2)	175 (4)
O1—H1*B*⋯F4^ii^	0.77 (4)	1.96 (4)	2.726 (2)	174 (3)
O2—H2*A*⋯O4	0.83 (3)	1.83 (3)	2.654 (2)	175 (3)
O2—H2*B*⋯F5	0.83 (4)	1.85 (4)	2.666 (2)	167 (3)
O3—H3*A*⋯F3^i^	0.84 (4)	1.85 (4)	2.683 (2)	171 (4)
O3—H3*B*⋯F6	0.90 (4)	1.81 (4)	2.683 (2)	163 (3)
O4—H4*A*⋯F3^iii^	0.75 (4)	2.00 (4)	2.718 (2)	163 (4)
O4—H4*B*⋯F2^i^	0.77 (3)	1.96 (3)	2.691 (2)	156 (3)

Symmetry codes: (i) 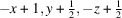; (ii) 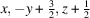; (iii) 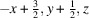.

**Table 2:** Hydrogen-bond geometry (Å, °) for (II)

*D*—H⋯*A*	*D*—H	H⋯*A*	*D*⋯*A*	*D*—H⋯*A*
O1—H1*A*⋯F5^i^	0.794 (18)	1.944 (18)	2.7338 (9)	173.5 (18)
O1—H1*B*⋯F4^ii^	0.78 (2)	1.93 (2)	2.7147 (10)	179 (2)
O2—H2*A*⋯F3^i^	0.79 (2)	1.85 (2)	2.6324 (10)	171 (2)
O2—H2*B*⋯F6	0.82 (2)	1.87 (2)	2.6491 (10)	159.2 (19)
O3—H3*A*⋯F2^iii^	0.79 (2)	1.85 (2)	2.6327 (10)	177.5 (19)
O3—H3*B*⋯O4^iv^	0.79 (2)	1.87 (2)	2.6481 (12)	170 (2)
O4—H4*A*⋯F3	0.799 (19)	2.002 (19)	2.7691 (10)	160.9 (18)
O4—H4*B*⋯F5^v^	0.78 (2)	2.02 (2)	2.7449 (11)	155 (2)

Symmetry codes: (i) 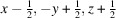; (ii) 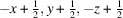; (iii) 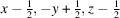; (iv) 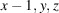; (v) 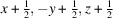.

**Table 3:** Hydrogen-bond geometry (Å, °) for (III)

*D*—H⋯*A*	*D*—H	H⋯*A*	*D*⋯*A*	*D*—H⋯*A*
O1—H1*A*⋯F5^i^	0.80 (3)	1.94 (3)	2.7359 (13)	172 (3)
O1—H1*B*⋯F4^ii^	0.77 (3)	1.95 (3)	2.7135 (13)	176 (3)
O2—H2*A*⋯F6	0.86 (3)	1.85 (3)	2.6456 (14)	154 (3)
O2—H2*B*⋯F3^i^	0.77 (3)	1.87 (3)	2.6362 (14)	171 (3)
O3—H3*A*⋯O4^iii^	0.81 (3)	1.85 (3)	2.6529 (17)	173 (3)
O3—H3*B*⋯F2^iv^	0.77 (3)	1.86 (3)	2.6330 (15)	176 (3)
O4—H4*A*⋯F5^v^	0.81 (3)	2.00 (3)	2.7429 (15)	154 (3)
O4—H4*B*⋯F3	0.81 (3)	2.01 (3)	2.7702 (14)	156 (3)

Symmetry codes: (i) 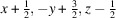; (ii) 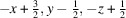; (iii) 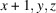; (iv) 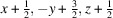; (v) 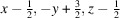.

**Table 4:** Hydrogen-bond geometry (Å, °) for (IV)

*D*—H⋯*A*	*D*—H	H⋯*A*	*D*⋯*A*	*D*—H⋯*A*
O1—H1*A*⋯F4	0.81 (3)	1.78 (3)	2.5926 (14)	176 (3)
O1—H1*B*⋯F1^i^	0.74 (3)	1.85 (3)	2.5861 (13)	172 (3)
O2—H2*A*⋯O3	0.74 (3)	1.95 (3)	2.6906 (15)	176 (3)
O2—H2*B*⋯F1^ii^	0.80 (3)	1.83 (3)	2.6255 (13)	175 (2)
O3—H3*A*⋯F2	0.78 (3)	1.94 (3)	2.7270 (17)	176 (3)
O3—H3*B*⋯F3^iii^	0.75 (3)	1.96 (3)	2.7020 (15)	173 (3)

Symmetry codes: (i) 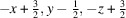; (ii) 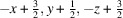; (iii) 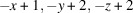.

**Table 5:** π–π stacking inter­actions in compounds (I)–(IV)

Compound number	type	*d*_phen­yl–pyridine_	*d*_pyridine–pyridine_	*d*_phen­yl–phen­yl_	inter­planar angle
(I)	face-to-face	3.699	4.162	3.583	0
(I)	displaced	6.042	4.128	8.111	8.68
(II)/(III)	parallel displaced	4.469	3.407	6.324	0
(II)/(III)	parallel displaced	3.510	4.472	4.035	0
(IV)	face-to-face	3.664	3.48	4.07	0
(IV)	parallel displaced	3.508	3.881	4.604	0

**Table 6:** Experimental details

	(I)	(II)	(III)	(IV)
Crystal data				
Chemical formula	[CuTiF_6_(C_12_H_8_N_2_)(H_2_O)_3_]·H_2_O	[CuZrF_6_(C_12_H_8_N_2_)(H_2_O)_3_]·H_2_O	[CuHfF_6_(C_12_H_8_N_2_)(H_2_O)_3_]·H_2_O	[CuF(C_12_H_8_N_2_)(H_2_O)_2_]_2_[HfF_6_]·2H_2_O
*M*_r_	477.71	521.03	608.30	926.07
Crystal system, space group	Orthorhombic, *P* *b* *c* *a*	Monoclinic, *P*2_1_/*n*	Monoclinic, *P*2_1_/*n*	Monoclinic, *P*2_1_/*n*
Temperature (K)	100	100	101	100
*a*, *b*, *c* (Å)	13.3603 (3), 14.1385 (3), 17.7895 (4)	9.9486 (4), 17.3006 (7), 10.0022 (4)	9.9411 (3), 17.2733 (4), 9.9972 (2)	13.6451 (2), 7.1161 (1), 15.7457 (3)
α, β, γ (°)	90, 90, 90	90, 95.1335 (18), 90	90, 95.116 (1), 90	90, 99.691 (1), 90
*V* (Å^3^)	3360.34 (13)	1714.64 (12)	1709.84 (7)	1507.09 (4)
*Z*	8	4	4	2
Radiation type	Mo *K*α	Mo *K*α	Mo *K*α	Mo *K*α
μ (mm^−1^)	1.83	1.93	7.39	4.93
Crystal size (mm)	0.09 × 0.07 × 0.05	0.24 × 0.12 × 0.11	0.17 × 0.12 × 0.05	0.16 × 0.16 × 0.10

Data collection				
Diffractometer	Bruker Kappa APEX CCD area detector	Bruker Kappa APEX CCD area detector	Bruker Kappa APEX CCD area detector	Bruker Kappa APEX CCD area detector
Absorption correction	Multi-scan (*SADABS*; Bruker, 2016 ▸)	Multi-scan (*SADABS*; Bruker, 2016 ▸)	Multi-scan (*SADABS*; Bruker, 2016 ▸)	Multi-scan (*SADABS*; Bruker, 2016 ▸)
*T*_min_, *T*_max_	0.668, 0.746	0.683, 0.747	0.480, 0.747	0.489, 0.746
No. of measured, independent and observed [*I* > 2σ(*I*)] reflections	36860, 4534, 3928	87607, 7546, 7052	43322, 8248, 7980	123138, 5034, 4982
*R*_int_	0.043	0.031	0.026	0.033
(sin θ/λ)_max_ (Å^−1^)	0.686	0.807	0.835	0.737

Refinement				
*R*[*F* ^2^ > 2σ(*F* ^2^)], *wR*(*F* ^2^), *S*	0.030, 0.071, 1.10	0.018, 0.047, 1.04	0.015, 0.036, 1.13	0.014, 0.035, 1.15
No. of reflections	4534	7546	8248	5034
No. of parameters	267	267	267	230
H-atom treatment	H atoms treated by a mixture of independent and constrained refinement	H atoms treated by a mixture of independent and constrained refinement	H atoms treated by a mixture of independent and constrained refinement	H atoms treated by a mixture of independent and constrained refinement
Δρ_max_, Δρ_min_ (e Å^−3^)	0.46, −0.45	0.57, −0.67	1.03, −0.98	0.67, −0.70

Computer programs: *APEX2* and *SAINT* (Bruker, 2017 ▸), *SHELXT* (Sheldrick, 2015*a* ▸), *SHELXL* (Sheldrick, 2015*b* ▸), and *OLEX2* (Dolomanov *et al.*, 2009 ▸).

## supplementary crystallographic information

### Triaqua-2κ3O-µ-fluorido-pentafluorido-1κ5F-(1,10-phenanthroline-2κ2N,N')copper(II)titanium(IV) monohydrate (I) Crystal data

**Table:**

[CuTiF_6_(C_12_H_8_N_2_)(H_2_O)_3_]·H_2_O	*D*_x_ = 1.889 Mg m^−^^3^
*M**_r_* = 477.71	Mo *K*α radiation, λ = 0.71073 Å
Orthorhombic, *P**b**c**a*	Cell parameters from 9952 reflections
*a* = 13.3603 (3) Å	θ = 2.8–29.1°
*b* = 14.1385 (3) Å	µ = 1.83 mm^−^^1^
*c* = 17.7895 (4) Å	*T* = 100 K
*V* = 3360.34 (13) Å^3^	Block, blue
*Z* = 8	0.09 × 0.07 × 0.05 mm
*F*(000) = 1912	

### Triaqua-2κ3O-µ-fluorido-pentafluorido-1κ5F-(1,10-phenanthroline-2κ2N,N')copper(II)titanium(IV) monohydrate (I) Data collection

**Table:**

Bruker Kappa APEX CCD area detector diffractometer	4534 independent reflections
Radiation source: sealed tube	3928 reflections with *I* > 2σ(*I*)
Triumph monochromator	*R*_int_ = 0.043
Detector resolution: 8 pixels mm^-1^	θ_max_ = 29.2°, θ_min_ = 2.3°
ω and φ scans	*h* = −18→18
Absorption correction: multi-scan (SADABS; Bruker, 2016)	*k* = −19→19
*T*_min_ = 0.668, *T*_max_ = 0.746	*l* = −23→24
36860 measured reflections	

### Triaqua-2κ3O-µ-fluorido-pentafluorido-1κ5F-(1,10-phenanthroline-2κ2N,N')copper(II)titanium(IV) monohydrate (I) Refinement

**Table:**

Refinement on *F*^2^	Primary atom site location: dual
Least-squares matrix: full	Hydrogen site location: mixed
*R*[*F*^2^ > 2σ(*F*^2^)] = 0.030	H atoms treated by a mixture of independent and constrained refinement
*wR*(*F*^2^) = 0.071	*w* = 1/[σ^2^(*F*_o_^2^) + (0.0163*P*)^2^ + 6.5495*P*] where *P* = (*F*_o_^2^ + 2*F*_c_^2^)/3
*S* = 1.10	(Δ/σ)_max_ = 0.001
4534 reflections	Δρ_max_ = 0.46 e Å^−^^3^
267 parameters	Δρ_min_ = −0.45 e Å^−^^3^
0 restraints	

### Triaqua-2κ3O-µ-fluorido-pentafluorido-1κ5F-(1,10-phenanthroline-2κ2N,N')copper(II)titanium(IV) monohydrate (I) Special details

**Table:**

Geometry. All esds (except the esd in the dihedral angle between two l.s. planes) are estimated using the full covariance matrix. The cell esds are taken into account individually in the estimation of esds in distances, angles and torsion angles; correlations between esds in cell parameters are only used when they are defined by crystal symmetry. An approximate (isotropic) treatment of cell esds is used for estimating esds involving l.s. planes.

### Triaqua-2κ3O-µ-fluorido-pentafluorido-1κ5F-(1,10-phenanthroline-2κ2N,N')copper(II)titanium(IV) monohydrate (I) Fractional atomic coordinates and isotropic or equivalent isotropic displacement parameters (Å^2^)

**Table:**

	*x*	*y*	*z*	*U*_iso_*/*U*_eq_	
Cu1	0.56746 (2)	0.74025 (2)	0.36021 (2)	0.01008 (7)	
Ti1	0.53873 (3)	0.60258 (3)	0.17156 (2)	0.00930 (8)	
F1	0.53330 (10)	0.61607 (9)	0.27499 (7)	0.0133 (2)	
F2	0.41521 (9)	0.54030 (9)	0.17056 (7)	0.0160 (3)	
F3	0.59857 (10)	0.48206 (9)	0.18751 (7)	0.0146 (3)	
F4	0.54844 (10)	0.58432 (9)	0.06857 (7)	0.0170 (3)	
F5	0.66406 (10)	0.65896 (10)	0.17257 (8)	0.0194 (3)	
F6	0.47531 (11)	0.71718 (9)	0.16027 (7)	0.0180 (3)	
O1	0.58746 (13)	0.88113 (12)	0.42069 (10)	0.0153 (3)	
H1A	0.586 (3)	0.923 (3)	0.400 (2)	0.036 (11)*	
H1B	0.578 (2)	0.894 (2)	0.462 (2)	0.033 (9)*	
O2	0.68191 (13)	0.76878 (12)	0.29363 (9)	0.0163 (3)	
H2A	0.702 (2)	0.824 (2)	0.2876 (17)	0.025 (8)*	
H2B	0.679 (3)	0.742 (2)	0.252 (2)	0.042 (10)*	
O3	0.46639 (13)	0.80398 (12)	0.29423 (9)	0.0160 (3)	
H3A	0.451 (3)	0.861 (3)	0.297 (2)	0.042 (10)*	
H3B	0.468 (3)	0.787 (2)	0.246 (2)	0.040 (10)*	
N1	0.46103 (13)	0.69042 (12)	0.42847 (10)	0.0105 (3)	
N2	0.65702 (13)	0.66919 (12)	0.43147 (10)	0.0109 (3)	
C1	0.36234 (16)	0.69370 (15)	0.42054 (12)	0.0129 (4)	
H1	0.334951	0.722036	0.376706	0.015*	
C2	0.29739 (16)	0.65669 (15)	0.47477 (13)	0.0149 (4)	
H2	0.227049	0.659406	0.467260	0.018*	
C3	0.33568 (17)	0.61635 (15)	0.53897 (13)	0.0150 (4)	
H3	0.292095	0.593534	0.577071	0.018*	
C4	0.44001 (17)	0.60926 (15)	0.54763 (12)	0.0136 (4)	
C5	0.49937 (16)	0.64734 (14)	0.48989 (12)	0.0105 (4)	
C6	0.48834 (18)	0.56371 (16)	0.60994 (13)	0.0166 (4)	
H6	0.449007	0.539269	0.649962	0.020*	
C7	0.58924 (18)	0.55511 (15)	0.61248 (12)	0.0166 (4)	
H7	0.619530	0.524951	0.654432	0.020*	
C8	0.65119 (17)	0.59062 (15)	0.55310 (12)	0.0146 (4)	
C9	0.60562 (16)	0.63669 (14)	0.49233 (12)	0.0109 (4)	
C10	0.75608 (18)	0.57771 (16)	0.54860 (13)	0.0176 (4)	
H10	0.791122	0.547634	0.588454	0.021*	
C11	0.80665 (17)	0.60883 (16)	0.48648 (14)	0.0179 (4)	
H11	0.876860	0.599295	0.482704	0.022*	
C12	0.75501 (16)	0.65480 (15)	0.42832 (13)	0.0145 (4)	
H12	0.791179	0.676164	0.385595	0.017*	
O4	0.74837 (14)	0.94525 (12)	0.28380 (11)	0.0194 (3)	
H4A	0.782 (3)	0.961 (2)	0.253 (2)	0.036 (10)*	
H4B	0.708 (2)	0.984 (2)	0.2893 (17)	0.026 (8)*	

### Triaqua-2κ3O-µ-fluorido-pentafluorido-1κ5F-(1,10-phenanthroline-2κ2N,N')copper(II)titanium(IV) monohydrate (I) Atomic displacement parameters (Å^2^)

**Table:**

	*U*^11^	*U*^22^	*U*^33^	*U*^12^	*U*^13^	*U*^23^
Cu1	0.01007 (12)	0.01075 (12)	0.00941 (12)	0.00031 (9)	0.00038 (9)	0.00084 (9)
Ti1	0.00972 (17)	0.00994 (16)	0.00822 (16)	−0.00046 (13)	0.00042 (13)	−0.00037 (13)
F1	0.0171 (6)	0.0134 (6)	0.0092 (6)	−0.0008 (5)	0.0015 (5)	−0.0002 (4)
F2	0.0106 (6)	0.0187 (6)	0.0188 (7)	−0.0023 (5)	0.0008 (5)	−0.0042 (5)
F3	0.0143 (6)	0.0134 (6)	0.0161 (6)	0.0024 (5)	−0.0012 (5)	−0.0010 (5)
F4	0.0198 (7)	0.0222 (7)	0.0088 (6)	0.0007 (5)	0.0013 (5)	−0.0017 (5)
F5	0.0159 (6)	0.0246 (7)	0.0176 (7)	−0.0090 (5)	0.0053 (5)	−0.0060 (5)
F6	0.0273 (7)	0.0125 (6)	0.0143 (6)	0.0049 (5)	−0.0031 (5)	0.0002 (5)
O1	0.0225 (9)	0.0130 (8)	0.0103 (8)	0.0006 (6)	0.0004 (6)	−0.0001 (6)
O2	0.0184 (8)	0.0168 (8)	0.0137 (8)	−0.0046 (6)	0.0044 (6)	−0.0022 (6)
O3	0.0212 (8)	0.0135 (8)	0.0132 (8)	0.0053 (6)	−0.0026 (6)	−0.0013 (6)
N1	0.0105 (8)	0.0103 (8)	0.0105 (8)	−0.0002 (6)	0.0007 (6)	−0.0008 (6)
N2	0.0097 (8)	0.0105 (8)	0.0126 (8)	0.0014 (6)	−0.0002 (6)	−0.0018 (6)
C1	0.0115 (10)	0.0145 (9)	0.0127 (10)	0.0011 (8)	−0.0016 (8)	−0.0021 (8)
C2	0.0106 (10)	0.0143 (10)	0.0198 (11)	−0.0003 (8)	0.0008 (8)	−0.0024 (8)
C3	0.0159 (10)	0.0143 (10)	0.0149 (10)	−0.0025 (8)	0.0051 (8)	−0.0035 (8)
C4	0.0169 (10)	0.0112 (9)	0.0126 (10)	−0.0005 (8)	0.0010 (8)	−0.0026 (7)
C5	0.0116 (9)	0.0095 (9)	0.0103 (9)	0.0001 (7)	−0.0004 (7)	−0.0018 (7)
C6	0.0244 (12)	0.0137 (10)	0.0116 (10)	−0.0007 (8)	0.0020 (9)	0.0006 (8)
C7	0.0250 (12)	0.0131 (10)	0.0116 (10)	0.0015 (8)	−0.0053 (9)	0.0015 (8)
C8	0.0179 (11)	0.0120 (9)	0.0138 (10)	0.0020 (8)	−0.0054 (8)	−0.0023 (8)
C9	0.0126 (10)	0.0088 (9)	0.0111 (9)	0.0010 (7)	−0.0007 (8)	−0.0018 (7)
C10	0.0178 (11)	0.0153 (10)	0.0196 (11)	0.0037 (8)	−0.0091 (9)	−0.0029 (8)
C11	0.0119 (10)	0.0175 (10)	0.0245 (12)	0.0042 (8)	−0.0043 (9)	−0.0032 (9)
C12	0.0121 (10)	0.0133 (9)	0.0180 (10)	−0.0001 (8)	−0.0003 (8)	−0.0046 (8)
O4	0.0165 (8)	0.0163 (8)	0.0255 (9)	0.0008 (7)	0.0078 (7)	0.0021 (7)

### Triaqua-2κ3O-µ-fluorido-pentafluorido-1κ5F-(1,10-phenanthroline-2κ2N,N')copper(II)titanium(IV) monohydrate (I) Geometric parameters (Å, º)

**Table:**

Cu1—F1	2.3643 (12)	C1—H1	0.9500
Cu1—O1	2.2794 (17)	C1—C2	1.399 (3)
Cu1—O2	1.9758 (16)	C2—H2	0.9500
Cu1—O3	2.0032 (16)	C2—C3	1.375 (3)
Cu1—N1	1.9981 (18)	C3—H3	0.9500
Cu1—N2	2.0120 (18)	C3—C4	1.406 (3)
Ti1—F1	1.8511 (13)	C4—C5	1.405 (3)
Ti1—F2	1.8706 (13)	C4—C6	1.435 (3)
Ti1—F3	1.9035 (13)	C5—C9	1.428 (3)
Ti1—F4	1.8548 (13)	C6—H6	0.9500
Ti1—F5	1.8545 (13)	C6—C7	1.354 (3)
Ti1—F6	1.8395 (13)	C7—H7	0.9500
O1—H1A	0.70 (4)	C7—C8	1.433 (3)
O1—H1B	0.77 (4)	C8—C9	1.401 (3)
O2—H2A	0.83 (3)	C8—C10	1.416 (3)
O2—H2B	0.83 (4)	C10—H10	0.9500
O3—H3A	0.84 (4)	C10—C11	1.368 (3)
O3—H3B	0.90 (4)	C11—H11	0.9500
N1—C1	1.327 (3)	C11—C12	1.403 (3)
N1—C5	1.352 (3)	C12—H12	0.9500
N2—C9	1.362 (3)	O4—H4A	0.75 (4)
N2—C12	1.326 (3)	O4—H4B	0.77 (3)
			
O1—Cu1—F1	166.96 (6)	C5—N1—Cu1	112.35 (14)
O2—Cu1—F1	85.21 (6)	C9—N2—Cu1	111.68 (14)
O2—Cu1—O1	90.79 (7)	C12—N2—Cu1	129.58 (15)
O2—Cu1—O3	94.51 (7)	C12—N2—C9	118.66 (19)
O2—Cu1—N1	170.75 (7)	N1—C1—H1	119.0
O2—Cu1—N2	91.11 (7)	N1—C1—C2	122.0 (2)
O3—Cu1—F1	80.11 (6)	C2—C1—H1	119.0
O3—Cu1—O1	87.85 (7)	C1—C2—H2	120.1
O3—Cu1—N2	174.07 (7)	C3—C2—C1	119.8 (2)
N1—Cu1—F1	89.45 (6)	C3—C2—H2	120.1
N1—Cu1—O1	96.01 (7)	C2—C3—H3	120.4
N1—Cu1—O3	92.01 (7)	C2—C3—C4	119.3 (2)
N1—Cu1—N2	82.20 (7)	C4—C3—H3	120.4
N2—Cu1—F1	98.51 (6)	C3—C4—C6	124.2 (2)
N2—Cu1—O1	93.97 (7)	C5—C4—C3	116.9 (2)
F1—Ti1—F2	91.34 (6)	C5—C4—C6	118.8 (2)
F1—Ti1—F3	87.74 (6)	N1—C5—C4	123.3 (2)
F1—Ti1—F4	177.26 (6)	N1—C5—C9	116.67 (19)
F1—Ti1—F5	88.94 (6)	C4—C5—C9	119.90 (19)
F2—Ti1—F3	87.17 (6)	C4—C6—H6	119.5
F4—Ti1—F2	89.24 (6)	C7—C6—C4	120.9 (2)
F4—Ti1—F3	89.61 (6)	C7—C6—H6	119.5
F5—Ti1—F2	177.38 (6)	C6—C7—H7	119.4
F5—Ti1—F3	90.24 (6)	C6—C7—C8	121.3 (2)
F5—Ti1—F4	90.36 (6)	C8—C7—H7	119.4
F6—Ti1—F1	89.99 (6)	C9—C8—C7	118.7 (2)
F6—Ti1—F2	90.41 (6)	C9—C8—C10	116.5 (2)
F6—Ti1—F3	176.64 (6)	C10—C8—C7	124.6 (2)
F6—Ti1—F4	92.69 (6)	N2—C9—C5	116.19 (18)
F6—Ti1—F5	92.20 (7)	N2—C9—C8	123.4 (2)
Ti1—F1—Cu1	134.93 (6)	C8—C9—C5	120.3 (2)
Cu1—O1—H1A	119 (3)	C8—C10—H10	120.2
Cu1—O1—H1B	130 (2)	C11—C10—C8	119.5 (2)
H1A—O1—H1B	107 (4)	C11—C10—H10	120.2
Cu1—O2—H2A	122 (2)	C10—C11—H11	119.9
Cu1—O2—H2B	113 (2)	C10—C11—C12	120.1 (2)
H2A—O2—H2B	109 (3)	C12—C11—H11	119.9
Cu1—O3—H3A	125 (2)	N2—C12—C11	121.7 (2)
Cu1—O3—H3B	115 (2)	N2—C12—H12	119.2
H3A—O3—H3B	109 (3)	C11—C12—H12	119.2
C1—N1—Cu1	129.07 (15)	H4A—O4—H4B	107 (3)
C1—N1—C5	118.57 (19)		

### Triaqua-2κ3O-µ-fluorido-pentafluorido-1κ5F-(1,10-phenanthroline-2κ2N,N')copper(II)titanium(IV) monohydrate (I) Hydrogen-bond geometry (Å, º)

**Table:**

*D*—H···*A*	*D*—H	H···*A*	*D*···*A*	*D*—H···*A*
O1—H1*A*···F2^i^	0.70 (4)	2.08 (4)	2.775 (2)	175 (4)
O1—H1*B*···F4^ii^	0.77 (4)	1.96 (4)	2.726 (2)	174 (3)
O2—H2*A*···O4	0.83 (3)	1.83 (3)	2.654 (2)	175 (3)
O2—H2*B*···F5	0.83 (4)	1.85 (4)	2.666 (2)	167 (3)
O3—H3*A*···F3^i^	0.84 (4)	1.85 (4)	2.683 (2)	171 (4)
O3—H3*B*···F6	0.90 (4)	1.81 (4)	2.683 (2)	163 (3)
O4—H4*A*···F3^iii^	0.75 (4)	2.00 (4)	2.718 (2)	163 (4)
O4—H4*B*···F2^i^	0.77 (3)	1.96 (3)	2.691 (2)	156 (3)

Symmetry codes: (i) −*x*+1, *y*+1/2, −*z*+1/2; (ii) *x*, −*y*+3/2, *z*+1/2; (iii) −*x*+3/2, *y*+1/2, *z*.

### Triaqua-2κ3O-µ-fluorido-pentafluorido-1κ5F-(1,10-phenanthroline-2κ2N,N')copper(II)zirconium(IV) monohydrate (II) Crystal data

**Table:**

[CuZrF_6_(C_12_H_8_N_2_)(H_2_O)_3_]·H_2_O	*F*(000) = 1028
*M**_r_* = 521.03	*D*_x_ = 2.018 Mg m^−^^3^
Monoclinic, *P*2_1_/*n*	Mo *K*α radiation, λ = 0.71073 Å
*a* = 9.9486 (4) Å	Cell parameters from 9425 reflections
*b* = 17.3006 (7) Å	θ = 3.0–34.9°
*c* = 10.0022 (4) Å	µ = 1.93 mm^−^^1^
β = 95.1335 (18)°	*T* = 100 K
*V* = 1714.64 (12) Å^3^	Cuboid, blue
*Z* = 4	0.24 × 0.12 × 0.11 mm

### Triaqua-2κ3O-µ-fluorido-pentafluorido-1κ5F-(1,10-phenanthroline-2κ2N,N')copper(II)zirconium(IV) monohydrate (II) Data collection

**Table:**

Bruker Kappa APEX CCD area detector diffractometer	7546 independent reflections
Radiation source: sealed tube	7052 reflections with *I* > 2σ(*I*)
Triumph monochromator	*R*_int_ = 0.031
Detector resolution: 8 pixels mm^-1^	θ_max_ = 35.0°, θ_min_ = 2.4°
ω and φ scans	*h* = −15→16
Absorption correction: multi-scan (SADABS; Bruker, 2016)	*k* = −27→27
*T*_min_ = 0.683, *T*_max_ = 0.747	*l* = −16→16
87607 measured reflections	

### Triaqua-2κ3O-µ-fluorido-pentafluorido-1κ5F-(1,10-phenanthroline-2κ2N,N')copper(II)zirconium(IV) monohydrate (II) Refinement

**Table:**

Refinement on *F*^2^	Primary atom site location: dual
Least-squares matrix: full	Hydrogen site location: mixed
*R*[*F*^2^ > 2σ(*F*^2^)] = 0.018	H atoms treated by a mixture of independent and constrained refinement
*wR*(*F*^2^) = 0.047	*w* = 1/[σ^2^(*F*_o_^2^) + (0.0222*P*)^2^ + 0.6601*P*] where *P* = (*F*_o_^2^ + 2*F*_c_^2^)/3
*S* = 1.04	(Δ/σ)_max_ = 0.001
7546 reflections	Δρ_max_ = 0.57 e Å^−^^3^
267 parameters	Δρ_min_ = −0.67 e Å^−^^3^
0 restraints	

### Triaqua-2κ3O-µ-fluorido-pentafluorido-1κ5F-(1,10-phenanthroline-2κ2N,N')copper(II)zirconium(IV) monohydrate (II) Special details

**Table:**

Geometry. All esds (except the esd in the dihedral angle between two l.s. planes) are estimated using the full covariance matrix. The cell esds are taken into account individually in the estimation of esds in distances, angles and torsion angles; correlations between esds in cell parameters are only used when they are defined by crystal symmetry. An approximate (isotropic) treatment of cell esds is used for estimating esds involving l.s. planes.

### Triaqua-2κ3O-µ-fluorido-pentafluorido-1κ5F-(1,10-phenanthroline-2κ2N,N')copper(II)zirconium(IV) monohydrate (II) Fractional atomic coordinates and isotropic or equivalent isotropic displacement parameters (Å^2^)

**Table:**

	*x*	*y*	*z*	*U*_iso_*/*U*_eq_	
Zr1	0.47531 (2)	0.17833 (2)	0.18511 (2)	0.00928 (2)	
Cu1	0.23490 (2)	0.36879 (2)	0.23859 (2)	0.01147 (3)	
F1	0.40575 (7)	0.28382 (3)	0.13913 (6)	0.01738 (11)	
F2	0.55807 (7)	0.20897 (4)	0.36687 (6)	0.02055 (12)	
F3	0.65583 (6)	0.20677 (4)	0.11564 (6)	0.01671 (11)	
F4	0.53575 (7)	0.06931 (3)	0.22042 (7)	0.01918 (11)	
F5	0.41618 (6)	0.14484 (4)	−0.00414 (6)	0.01543 (10)	
F6	0.29772 (6)	0.15382 (4)	0.25277 (6)	0.01807 (11)	
O1	0.09615 (8)	0.43805 (4)	0.36235 (8)	0.01672 (13)	
H1A	0.0425 (17)	0.4171 (10)	0.4041 (17)	0.028 (4)*	
H1B	0.0583 (19)	0.4762 (12)	0.3397 (19)	0.039 (5)*	
O2	0.22635 (9)	0.28333 (4)	0.36875 (8)	0.02195 (15)	
H2A	0.198 (2)	0.2842 (11)	0.440 (2)	0.043 (5)*	
H2B	0.2341 (19)	0.2387 (11)	0.3444 (19)	0.039 (5)*	
O3	0.08658 (9)	0.32098 (6)	0.12593 (9)	0.02716 (19)	
H3A	0.0786 (18)	0.3133 (10)	0.048 (2)	0.034 (5)*	
H3B	0.019 (2)	0.3082 (11)	0.155 (2)	0.040 (5)*	
N1	0.40931 (8)	0.40912 (4)	0.32659 (8)	0.01241 (12)	
N2	0.25539 (8)	0.45780 (4)	0.11410 (7)	0.01176 (12)	
C1	0.48666 (10)	0.38078 (6)	0.42972 (10)	0.01668 (16)	
H1	0.456184	0.336879	0.475402	0.020*	
C2	0.61195 (11)	0.41331 (6)	0.47385 (10)	0.02059 (19)	
H2	0.665231	0.391295	0.547766	0.025*	
C3	0.65728 (10)	0.47718 (7)	0.40963 (11)	0.02061 (19)	
H3	0.742169	0.499549	0.438437	0.025*	
C4	0.57629 (9)	0.50917 (6)	0.30041 (10)	0.01582 (16)	
C5	0.45370 (9)	0.47190 (5)	0.26198 (9)	0.01215 (14)	
C6	0.61134 (10)	0.57674 (6)	0.22755 (11)	0.02057 (18)	
H6	0.693485	0.602912	0.253555	0.025*	
C7	0.52933 (11)	0.60382 (6)	0.12238 (11)	0.01943 (18)	
H7	0.554529	0.648907	0.076505	0.023*	
C8	0.40516 (10)	0.56548 (5)	0.07928 (9)	0.01432 (15)	
C9	0.36882 (9)	0.49938 (5)	0.14917 (9)	0.01139 (13)	
C10	0.31652 (11)	0.58914 (6)	−0.03080 (10)	0.01766 (17)	
H10	0.336075	0.633809	−0.080793	0.021*	
C11	0.20132 (11)	0.54697 (6)	−0.06530 (10)	0.01771 (16)	
H11	0.140492	0.562526	−0.139020	0.021*	
C12	0.17425 (9)	0.48094 (6)	0.00892 (9)	0.01485 (15)	
H12	0.095257	0.451756	−0.016796	0.018*	
O4	0.86608 (9)	0.29013 (6)	0.24604 (9)	0.02461 (17)	
H4A	0.8042 (19)	0.2617 (10)	0.2257 (18)	0.031 (4)*	
H4B	0.864 (2)	0.2988 (12)	0.322 (2)	0.042 (5)*	

### Triaqua-2κ3O-µ-fluorido-pentafluorido-1κ5F-(1,10-phenanthroline-2κ2N,N')copper(II)zirconium(IV) monohydrate (II) Atomic displacement parameters (Å^2^)

**Table:**

	*U*^11^	*U*^22^	*U*^33^	*U*^12^	*U*^13^	*U*^23^
Zr1	0.01037 (4)	0.00955 (4)	0.00795 (4)	−0.00034 (2)	0.00102 (3)	−0.00012 (2)
Cu1	0.01226 (5)	0.01299 (5)	0.00913 (5)	−0.00278 (3)	0.00070 (4)	0.00011 (3)
F1	0.0245 (3)	0.0125 (2)	0.0153 (2)	0.0038 (2)	0.0034 (2)	0.00169 (19)
F2	0.0276 (3)	0.0226 (3)	0.0105 (2)	−0.0005 (2)	−0.0032 (2)	−0.0021 (2)
F3	0.0134 (2)	0.0231 (3)	0.0138 (2)	−0.0050 (2)	0.0024 (2)	−0.0008 (2)
F4	0.0211 (3)	0.0117 (2)	0.0239 (3)	0.0020 (2)	−0.0028 (2)	0.0013 (2)
F5	0.0146 (2)	0.0202 (3)	0.0111 (2)	0.0012 (2)	−0.00087 (19)	−0.00385 (19)
F6	0.0169 (3)	0.0176 (3)	0.0209 (3)	−0.0025 (2)	0.0084 (2)	−0.0003 (2)
O1	0.0169 (3)	0.0148 (3)	0.0194 (3)	0.0016 (2)	0.0069 (3)	0.0026 (2)
O2	0.0385 (5)	0.0138 (3)	0.0155 (3)	0.0005 (3)	0.0134 (3)	0.0004 (2)
O3	0.0223 (4)	0.0459 (5)	0.0138 (3)	−0.0194 (4)	0.0047 (3)	−0.0101 (3)
N1	0.0129 (3)	0.0130 (3)	0.0111 (3)	0.0020 (2)	−0.0005 (2)	−0.0016 (2)
N2	0.0099 (3)	0.0147 (3)	0.0107 (3)	−0.0003 (2)	0.0012 (2)	0.0001 (2)
C1	0.0184 (4)	0.0180 (4)	0.0130 (4)	0.0059 (3)	−0.0024 (3)	−0.0022 (3)
C2	0.0165 (4)	0.0273 (5)	0.0167 (4)	0.0079 (4)	−0.0055 (3)	−0.0057 (4)
C3	0.0118 (4)	0.0282 (5)	0.0209 (4)	0.0019 (3)	−0.0030 (3)	−0.0099 (4)
C4	0.0110 (4)	0.0187 (4)	0.0177 (4)	−0.0007 (3)	0.0011 (3)	−0.0068 (3)
C5	0.0104 (3)	0.0136 (3)	0.0124 (3)	0.0002 (3)	0.0006 (3)	−0.0034 (3)
C6	0.0149 (4)	0.0201 (4)	0.0273 (5)	−0.0064 (3)	0.0053 (4)	−0.0079 (4)
C7	0.0193 (4)	0.0153 (4)	0.0248 (5)	−0.0050 (3)	0.0083 (4)	−0.0034 (3)
C8	0.0161 (4)	0.0118 (3)	0.0157 (4)	−0.0007 (3)	0.0054 (3)	−0.0014 (3)
C9	0.0103 (3)	0.0125 (3)	0.0116 (3)	0.0002 (2)	0.0023 (3)	−0.0013 (3)
C10	0.0238 (5)	0.0140 (4)	0.0159 (4)	0.0026 (3)	0.0058 (3)	0.0021 (3)
C11	0.0208 (4)	0.0189 (4)	0.0133 (4)	0.0051 (3)	0.0007 (3)	0.0030 (3)
C12	0.0129 (4)	0.0188 (4)	0.0125 (3)	0.0017 (3)	−0.0005 (3)	0.0011 (3)
O4	0.0210 (4)	0.0353 (4)	0.0176 (3)	−0.0141 (3)	0.0026 (3)	−0.0055 (3)

### Triaqua-2κ3O-µ-fluorido-pentafluorido-1κ5F-(1,10-phenanthroline-2κ2N,N')copper(II)zirconium(IV) monohydrate (II) Geometric parameters (Å, º)

**Table:**

Zr1—F1	1.9910 (6)	C1—H1	0.9500
Zr1—F2	1.9991 (6)	C1—C2	1.4023 (15)
Zr1—F3	2.0430 (6)	C2—H2	0.9500
Zr1—F4	2.0014 (6)	C2—C3	1.3744 (17)
Zr1—F5	2.0163 (6)	C3—H3	0.9500
Zr1—F6	1.9933 (6)	C3—C4	1.4111 (15)
Cu1—F1	2.5184 (6)	C4—C5	1.4025 (13)
Cu1—O1	2.2758 (7)	C4—C6	1.4369 (15)
Cu1—O2	1.9768 (8)	C5—C9	1.4288 (13)
Cu1—O3	1.9580 (8)	C6—H6	0.9500
Cu1—N1	1.9997 (8)	C6—C7	1.3558 (17)
Cu1—N2	2.0021 (8)	C7—H7	0.9500
O1—H1A	0.794 (18)	C7—C8	1.4341 (14)
O1—H1B	0.78 (2)	C8—C9	1.4044 (12)
O2—H2A	0.79 (2)	C8—C10	1.4085 (14)
O2—H2B	0.82 (2)	C10—H10	0.9500
O3—H3A	0.79 (2)	C10—C11	1.3762 (15)
O3—H3B	0.79 (2)	C11—H11	0.9500
N1—C1	1.3245 (12)	C11—C12	1.4015 (13)
N1—C5	1.3577 (12)	C12—H12	0.9500
N2—C9	1.3577 (11)	O4—H4A	0.799 (19)
N2—C12	1.3292 (12)	O4—H4B	0.78 (2)
			
F1—Zr1—F2	94.21 (3)	C5—N1—Cu1	112.08 (6)
F1—Zr1—F3	89.92 (3)	C9—N2—Cu1	112.05 (6)
F1—Zr1—F4	175.79 (3)	C12—N2—Cu1	129.46 (6)
F1—Zr1—F5	88.87 (3)	C12—N2—C9	118.47 (8)
F1—Zr1—F6	88.46 (3)	N1—C1—H1	118.8
F2—Zr1—F3	86.70 (3)	N1—C1—C2	122.32 (10)
F2—Zr1—F4	89.81 (3)	C2—C1—H1	118.8
F2—Zr1—F5	172.66 (3)	C1—C2—H2	120.2
F4—Zr1—F3	91.58 (3)	C3—C2—C1	119.67 (9)
F4—Zr1—F5	87.29 (3)	C3—C2—H2	120.2
F5—Zr1—F3	86.64 (2)	C2—C3—H3	120.4
F6—Zr1—F2	93.04 (3)	C2—C3—C4	119.25 (9)
F6—Zr1—F3	178.34 (3)	C4—C3—H3	120.4
F6—Zr1—F4	90.06 (3)	C3—C4—C6	124.33 (9)
F6—Zr1—F5	93.71 (3)	C5—C4—C3	117.02 (9)
O1—Cu1—F1	170.35 (2)	C5—C4—C6	118.65 (9)
O2—Cu1—F1	83.91 (3)	N1—C5—C4	123.31 (9)
O2—Cu1—O1	88.37 (3)	N1—C5—C9	116.59 (8)
O2—Cu1—N1	93.32 (4)	C4—C5—C9	120.10 (8)
O2—Cu1—N2	176.00 (4)	C4—C6—H6	119.4
O3—Cu1—F1	91.52 (3)	C7—C6—C4	121.21 (9)
O3—Cu1—O1	94.18 (3)	C7—C6—H6	119.4
O3—Cu1—O2	89.34 (4)	C6—C7—H7	119.5
O3—Cu1—N1	168.59 (3)	C6—C7—C8	121.05 (9)
O3—Cu1—N2	94.61 (4)	C8—C7—H7	119.5
N1—Cu1—F1	77.75 (3)	C9—C8—C7	118.70 (9)
N1—Cu1—O1	96.98 (3)	C9—C8—C10	117.05 (9)
N1—Cu1—N2	82.69 (3)	C10—C8—C7	124.25 (9)
N2—Cu1—F1	95.36 (3)	N2—C9—C5	116.52 (8)
N2—Cu1—O1	91.94 (3)	N2—C9—C8	123.22 (8)
Zr1—F1—Cu1	132.59 (3)	C8—C9—C5	120.25 (8)
Cu1—O1—H1A	121.0 (13)	C8—C10—H10	120.3
Cu1—O1—H1B	125.9 (14)	C11—C10—C8	119.41 (9)
H1A—O1—H1B	102.0 (18)	C11—C10—H10	120.3
Cu1—O2—H2A	128.2 (14)	C10—C11—H11	120.2
Cu1—O2—H2B	120.1 (13)	C10—C11—C12	119.63 (9)
H2A—O2—H2B	109.8 (19)	C12—C11—H11	120.2
Cu1—O3—H3A	130.1 (13)	N2—C12—C11	122.20 (9)
Cu1—O3—H3B	122.1 (15)	N2—C12—H12	118.9
H3A—O3—H3B	107.6 (19)	C11—C12—H12	118.9
C1—N1—Cu1	129.37 (7)	H4A—O4—H4B	106.4 (19)
C1—N1—C5	118.41 (8)		

### Triaqua-2κ3O-µ-fluorido-pentafluorido-1κ5F-(1,10-phenanthroline-2κ2N,N')copper(II)zirconium(IV) monohydrate (II) Hydrogen-bond geometry (Å, º)

**Table:**

*D*—H···*A*	*D*—H	H···*A*	*D*···*A*	*D*—H···*A*
O1—H1*A*···F5^i^	0.794 (18)	1.944 (18)	2.7338 (9)	173.5 (18)
O1—H1*B*···F4^ii^	0.78 (2)	1.93 (2)	2.7147 (10)	179 (2)
O2—H2*A*···F3^i^	0.79 (2)	1.85 (2)	2.6324 (10)	171 (2)
O2—H2*B*···F6	0.82 (2)	1.87 (2)	2.6491 (10)	159.2 (19)
O3—H3*A*···F2^iii^	0.79 (2)	1.85 (2)	2.6327 (10)	177.5 (19)
O3—H3*B*···O4^iv^	0.79 (2)	1.87 (2)	2.6481 (12)	170 (2)
O4—H4*A*···F3	0.799 (19)	2.002 (19)	2.7691 (10)	160.9 (18)
O4—H4*B*···F5^v^	0.78 (2)	2.02 (2)	2.7449 (11)	155 (2)

Symmetry codes: (i) *x*−1/2, −*y*+1/2, *z*+1/2; (ii) −*x*+1/2, *y*+1/2, −*z*+1/2; (iii) *x*−1/2, −*y*+1/2, *z*−1/2; (iv) *x*−1, *y*, *z*; (v) *x*+1/2, −*y*+1/2, *z*+1/2.

### Triaqua-2κ3O-µ-fluorido-pentafluorido-1κ5F-(1,10-phenanthroline-2κ2N,N')copper(II)hafnium(IV) monohydrate (III) Crystal data

**Table:**

[CuHfF_6_(C_12_H_8_N_2_)(H_2_O)_3_]·H_2_O	*F*(000) = 1156
*M**_r_* = 608.30	*D*_x_ = 2.363 Mg m^−^^3^
Monoclinic, *P*2_1_/*n*	Mo *K*α radiation, λ = 0.71073 Å
*a* = 9.9411 (3) Å	Cell parameters from 9921 reflections
*b* = 17.2733 (4) Å	θ = 2.4–36.4°
*c* = 9.9972 (2) Å	µ = 7.39 mm^−^^1^
β = 95.116 (1)°	*T* = 101 K
*V* = 1709.84 (7) Å^3^	Rodlike, blue
*Z* = 4	0.17 × 0.12 × 0.05 mm

### Triaqua-2κ3O-µ-fluorido-pentafluorido-1κ5F-(1,10-phenanthroline-2κ2N,N')copper(II)hafnium(IV) monohydrate (III) Data collection

**Table:**

Bruker Kappa APEX CCD area detector diffractometer	8248 independent reflections
Radiation source: sealed tube	7980 reflections with *I* > 2σ(*I*)
Triumph monochromator	*R*_int_ = 0.026
Detector resolution: 8 pixels mm^-1^	θ_max_ = 36.4°, θ_min_ = 2.4°
ω and φ scans	*h* = −16→16
Absorption correction: multi-scan (SADABS; Bruker, 2016)	*k* = −28→28
*T*_min_ = 0.480, *T*_max_ = 0.747	*l* = −13→16
43322 measured reflections	

### Triaqua-2κ3O-µ-fluorido-pentafluorido-1κ5F-(1,10-phenanthroline-2κ2N,N')copper(II)hafnium(IV) monohydrate (III) Refinement

**Table:**

Refinement on *F*^2^	Primary atom site location: dual
Least-squares matrix: full	Hydrogen site location: mixed
*R*[*F*^2^ > 2σ(*F*^2^)] = 0.015	H atoms treated by a mixture of independent and constrained refinement
*wR*(*F*^2^) = 0.036	*w* = 1/[σ^2^(*F*_o_^2^) + (0.0108*P*)^2^ + 0.940*P*] where *P* = (*F*_o_^2^ + 2*F*_c_^2^)/3
*S* = 1.13	(Δ/σ)_max_ = 0.005
8248 reflections	Δρ_max_ = 1.03 e Å^−^^3^
267 parameters	Δρ_min_ = −0.98 e Å^−^^3^
0 restraints	

### Triaqua-2κ3O-µ-fluorido-pentafluorido-1κ5F-(1,10-phenanthroline-2κ2N,N')copper(II)hafnium(IV) monohydrate (III) Special details

**Table:**

Geometry. All esds (except the esd in the dihedral angle between two l.s. planes) are estimated using the full covariance matrix. The cell esds are taken into account individually in the estimation of esds in distances, angles and torsion angles; correlations between esds in cell parameters are only used when they are defined by crystal symmetry. An approximate (isotropic) treatment of cell esds is used for estimating esds involving l.s. planes.

### Triaqua-2κ3O-µ-fluorido-pentafluorido-1κ5F-(1,10-phenanthroline-2κ2N,N')copper(II)hafnium(IV) monohydrate (III) Fractional atomic coordinates and isotropic or equivalent isotropic displacement parameters (Å^2^)

**Table:**

	*x*	*y*	*z*	*U*_iso_*/*U*_eq_	
Hf1	0.52483 (2)	0.82180 (2)	0.31483 (2)	0.00816 (1)	
Cu1	0.76520 (2)	0.63146 (2)	0.26150 (2)	0.01043 (2)	
F1	0.59437 (9)	0.71639 (5)	0.36048 (9)	0.01579 (14)	
F2	0.44315 (10)	0.79101 (5)	0.13341 (9)	0.01846 (15)	
F3	0.34510 (8)	0.79333 (5)	0.38379 (9)	0.01545 (13)	
F4	0.46393 (9)	0.93051 (5)	0.27960 (10)	0.01710 (15)	
F5	0.58415 (8)	0.85522 (5)	0.50333 (8)	0.01413 (13)	
F6	0.70198 (9)	0.84641 (5)	0.24732 (9)	0.01652 (14)	
O1	0.90410 (10)	0.56191 (6)	0.13766 (11)	0.01534 (16)	
H1A	0.952 (3)	0.5850 (18)	0.092 (3)	0.043 (8)*	
H1B	0.944 (3)	0.5250 (18)	0.158 (3)	0.038 (8)*	
O2	0.77370 (14)	0.71712 (6)	0.13085 (11)	0.0204 (2)	
H2A	0.770 (3)	0.7646 (16)	0.155 (3)	0.039 (8)*	
H2B	0.794 (3)	0.7190 (18)	0.058 (3)	0.041 (8)*	
O3	0.91370 (13)	0.67963 (8)	0.37400 (13)	0.0258 (3)	
H3A	0.982 (3)	0.6915 (16)	0.342 (3)	0.036 (8)*	
H3B	0.923 (3)	0.6862 (16)	0.451 (3)	0.036 (8)*	
N1	0.59097 (11)	0.59097 (6)	0.17348 (10)	0.01118 (15)	
N2	0.74478 (10)	0.54241 (6)	0.38597 (10)	0.01065 (14)	
C1	0.51346 (13)	0.61947 (7)	0.07036 (13)	0.01500 (19)	
H1	0.543886	0.663510	0.024768	0.018*	
C2	0.38773 (14)	0.58678 (9)	0.02611 (15)	0.0187 (2)	
H2	0.334281	0.608849	−0.047718	0.022*	
C3	0.34248 (13)	0.52246 (9)	0.09056 (15)	0.0185 (2)	
H3	0.257713	0.499831	0.061741	0.022*	
C4	0.42372 (12)	0.49081 (7)	0.19968 (14)	0.01433 (19)	
C5	0.38832 (14)	0.42310 (8)	0.27286 (16)	0.0186 (2)	
H5	0.305923	0.397016	0.247140	0.022*	
C6	0.47075 (14)	0.39590 (8)	0.37801 (15)	0.0175 (2)	
H6	0.445833	0.350595	0.423706	0.021*	
C7	0.59489 (13)	0.43449 (7)	0.42121 (13)	0.01293 (18)	
C8	0.63154 (11)	0.50068 (6)	0.35078 (12)	0.01037 (16)	
C9	0.54650 (11)	0.52808 (7)	0.23831 (12)	0.01084 (16)	
C10	0.68394 (14)	0.41075 (7)	0.53102 (14)	0.0160 (2)	
H10	0.664685	0.365860	0.580801	0.019*	
C11	0.79903 (14)	0.45307 (7)	0.56563 (14)	0.0159 (2)	
H11	0.859827	0.437608	0.639508	0.019*	
C12	0.82601 (12)	0.51916 (7)	0.49119 (13)	0.01334 (18)	
H12	0.905102	0.548379	0.516812	0.016*	
O4	0.13489 (12)	0.70967 (8)	0.25315 (12)	0.0224 (2)	
H4A	0.143 (3)	0.6980 (16)	0.176 (3)	0.030 (7)*	
H4B	0.200 (3)	0.7376 (18)	0.269 (3)	0.043 (8)*	

### Triaqua-2κ3O-µ-fluorido-pentafluorido-1κ5F-(1,10-phenanthroline-2κ2N,N')copper(II)hafnium(IV) monohydrate (III) Atomic displacement parameters (Å^2^)

**Table:**

	*U*^11^	*U*^22^	*U*^33^	*U*^12^	*U*^13^	*U*^23^
Hf1	0.00935 (2)	0.00804 (2)	0.00717 (2)	−0.00036 (1)	0.00110 (1)	−0.00014 (1)
Cu1	0.01135 (5)	0.01143 (5)	0.00851 (6)	−0.00251 (4)	0.00096 (4)	0.00007 (4)
F1	0.0220 (4)	0.0106 (3)	0.0152 (3)	0.0038 (3)	0.0040 (3)	0.0018 (2)
F2	0.0247 (4)	0.0205 (4)	0.0095 (3)	−0.0001 (3)	−0.0024 (3)	−0.0022 (3)
F3	0.0121 (3)	0.0211 (4)	0.0134 (3)	−0.0043 (3)	0.0027 (2)	−0.0008 (3)
F4	0.0185 (4)	0.0101 (3)	0.0220 (4)	0.0021 (2)	−0.0022 (3)	0.0012 (3)
F5	0.0138 (3)	0.0182 (3)	0.0101 (3)	0.0009 (2)	−0.0005 (2)	−0.0036 (2)
F6	0.0154 (3)	0.0159 (3)	0.0195 (4)	−0.0027 (3)	0.0086 (3)	−0.0005 (3)
O1	0.0153 (4)	0.0138 (4)	0.0177 (4)	0.0018 (3)	0.0062 (3)	0.0026 (3)
O2	0.0361 (6)	0.0122 (4)	0.0147 (4)	0.0008 (4)	0.0127 (4)	0.0002 (3)
O3	0.0208 (5)	0.0440 (7)	0.0132 (5)	−0.0190 (5)	0.0050 (4)	−0.0095 (4)
N1	0.0121 (4)	0.0118 (4)	0.0094 (4)	0.0016 (3)	−0.0002 (3)	−0.0011 (3)
N2	0.0094 (3)	0.0127 (4)	0.0098 (4)	−0.0003 (3)	0.0005 (3)	0.0000 (3)
C1	0.0168 (5)	0.0155 (5)	0.0120 (5)	0.0052 (4)	−0.0023 (4)	−0.0018 (3)
C2	0.0141 (5)	0.0248 (6)	0.0160 (5)	0.0063 (4)	−0.0045 (4)	−0.0049 (4)
C3	0.0112 (4)	0.0253 (6)	0.0183 (6)	0.0016 (4)	−0.0025 (4)	−0.0082 (4)
C4	0.0103 (4)	0.0164 (5)	0.0162 (5)	−0.0012 (3)	0.0009 (3)	−0.0056 (4)
C5	0.0137 (5)	0.0184 (5)	0.0242 (6)	−0.0060 (4)	0.0052 (4)	−0.0060 (4)
C6	0.0178 (5)	0.0138 (5)	0.0221 (6)	−0.0052 (4)	0.0073 (4)	−0.0030 (4)
C7	0.0152 (4)	0.0100 (4)	0.0143 (5)	−0.0003 (3)	0.0051 (4)	−0.0007 (3)
C8	0.0096 (4)	0.0105 (4)	0.0113 (4)	−0.0008 (3)	0.0025 (3)	−0.0011 (3)
C9	0.0090 (4)	0.0123 (4)	0.0111 (4)	0.0002 (3)	0.0007 (3)	−0.0026 (3)
C10	0.0212 (5)	0.0127 (4)	0.0148 (5)	0.0024 (4)	0.0053 (4)	0.0022 (4)
C11	0.0190 (5)	0.0158 (5)	0.0129 (5)	0.0038 (4)	0.0008 (4)	0.0032 (4)
C12	0.0121 (4)	0.0166 (5)	0.0110 (4)	0.0010 (3)	−0.0004 (3)	0.0012 (3)
O4	0.0190 (4)	0.0316 (6)	0.0167 (5)	−0.0120 (4)	0.0027 (3)	−0.0048 (4)

### Triaqua-2κ3O-µ-fluorido-pentafluorido-1κ5F-(1,10-phenanthroline-2κ2N,N')copper(II)hafnium(IV) monohydrate (III) Geometric parameters (Å, º)

**Table:**

Hf1—F1	1.9863 (8)	C1—H1	0.9500
Hf1—F2	1.9922 (9)	C1—C2	1.4065 (19)
Hf1—F3	2.0320 (8)	C2—H2	0.9500
Hf1—F4	1.9946 (8)	C2—C3	1.380 (2)
Hf1—F5	2.0085 (8)	C3—H3	0.9500
Hf1—F6	1.9873 (8)	C3—C4	1.409 (2)
Cu1—F1	2.5130 (9)	C4—C5	1.440 (2)
Cu1—O1	2.2776 (10)	C4—C9	1.4033 (16)
Cu1—O2	1.9804 (11)	C5—H5	0.9500
Cu1—O3	1.9597 (11)	C5—C6	1.358 (2)
Cu1—N1	1.9979 (11)	C6—H6	0.9500
Cu1—N2	2.0002 (10)	C6—C7	1.4348 (18)
O1—H1A	0.80 (3)	C7—C8	1.4076 (17)
O1—H1B	0.77 (3)	C7—C10	1.4085 (19)
O2—H2A	0.86 (3)	C8—C9	1.4259 (17)
O2—H2B	0.77 (3)	C10—H10	0.9500
O3—H3A	0.81 (3)	C10—C11	1.376 (2)
O3—H3B	0.77 (3)	C11—H11	0.9500
N1—C1	1.3256 (16)	C11—C12	1.4016 (18)
N1—C9	1.3590 (16)	C12—H12	0.9500
N2—C8	1.3565 (15)	O4—H4A	0.81 (3)
N2—C12	1.3298 (16)	O4—H4B	0.81 (3)
			
F1—Hf1—F2	94.00 (4)	C9—N1—Cu1	112.08 (8)
F1—Hf1—F3	89.93 (4)	C8—N2—Cu1	112.04 (8)
F1—Hf1—F4	175.95 (4)	C12—N2—Cu1	129.51 (8)
F1—Hf1—F5	88.90 (4)	C12—N2—C8	118.43 (10)
F1—Hf1—F6	88.47 (4)	N1—C1—H1	118.9
F2—Hf1—F3	86.85 (4)	N1—C1—C2	122.28 (13)
F2—Hf1—F4	89.88 (4)	C2—C1—H1	118.9
F2—Hf1—F5	173.04 (4)	C1—C2—H2	120.2
F4—Hf1—F3	91.46 (4)	C3—C2—C1	119.61 (12)
F4—Hf1—F5	87.38 (4)	C3—C2—H2	120.2
F5—Hf1—F3	86.83 (3)	C2—C3—H3	120.5
F6—Hf1—F2	92.84 (4)	C2—C3—C4	119.07 (12)
F6—Hf1—F3	178.34 (4)	C4—C3—H3	120.5
F6—Hf1—F4	90.17 (4)	C3—C4—C5	124.03 (12)
F6—Hf1—F5	93.57 (4)	C9—C4—C3	117.37 (12)
O1—Cu1—F1	170.30 (3)	C9—C4—C5	118.61 (12)
O2—Cu1—F1	83.89 (4)	C4—C5—H5	119.4
O2—Cu1—O1	88.40 (4)	C6—C5—C4	121.10 (12)
O2—Cu1—N1	93.29 (5)	C6—C5—H5	119.4
O2—Cu1—N2	175.97 (5)	C5—C6—H6	119.5
O3—Cu1—F1	91.55 (5)	C5—C6—C7	121.04 (12)
O3—Cu1—O1	94.23 (5)	C7—C6—H6	119.5
O3—Cu1—O2	89.26 (6)	C8—C7—C6	118.74 (12)
O3—Cu1—N1	168.66 (5)	C8—C7—C10	116.98 (11)
O3—Cu1—N2	94.73 (5)	C10—C7—C6	124.28 (12)
N1—Cu1—F1	77.76 (4)	N2—C8—C7	123.21 (11)
N1—Cu1—O1	96.88 (4)	N2—C8—C9	116.62 (10)
N1—Cu1—N2	82.69 (4)	C7—C8—C9	120.16 (10)
N2—Cu1—F1	95.41 (4)	N1—C9—C4	123.19 (11)
N2—Cu1—O1	91.87 (4)	N1—C9—C8	116.50 (10)
Hf1—F1—Cu1	132.71 (4)	C4—C9—C8	120.31 (11)
Cu1—O1—H1A	118 (2)	C7—C10—H10	120.3
Cu1—O1—H1B	128 (2)	C11—C10—C7	119.48 (12)
H1A—O1—H1B	104 (3)	C11—C10—H10	120.3
Cu1—O2—H2A	121 (2)	C10—C11—H11	120.2
Cu1—O2—H2B	133 (2)	C10—C11—C12	119.57 (12)
H2A—O2—H2B	104 (3)	C12—C11—H11	120.2
Cu1—O3—H3A	120 (2)	N2—C12—C11	122.31 (12)
Cu1—O3—H3B	131 (2)	N2—C12—H12	118.8
H3A—O3—H3B	109 (3)	C11—C12—H12	118.8
C1—N1—Cu1	129.31 (9)	H4A—O4—H4B	101 (3)
C1—N1—C9	118.46 (11)		

### Triaqua-2κ3O-µ-fluorido-pentafluorido-1κ5F-(1,10-phenanthroline-2κ2N,N')copper(II)hafnium(IV) monohydrate (III) Hydrogen-bond geometry (Å, º)

**Table:**

*D*—H···*A*	*D*—H	H···*A*	*D*···*A*	*D*—H···*A*
O1—H1*A*···F5^i^	0.80 (3)	1.94 (3)	2.7359 (13)	172 (3)
O1—H1*B*···F4^ii^	0.77 (3)	1.95 (3)	2.7135 (13)	176 (3)
O2—H2*A*···F6	0.86 (3)	1.85 (3)	2.6456 (14)	154 (3)
O2—H2*B*···F3^i^	0.77 (3)	1.87 (3)	2.6362 (14)	171 (3)
O3—H3*A*···O4^iii^	0.81 (3)	1.85 (3)	2.6529 (17)	173 (3)
O3—H3*B*···F2^iv^	0.77 (3)	1.86 (3)	2.6330 (15)	176 (3)
O4—H4*A*···F5^v^	0.81 (3)	2.00 (3)	2.7429 (15)	154 (3)
O4—H4*B*···F3	0.81 (3)	2.01 (3)	2.7702 (14)	156 (3)

Symmetry codes: (i) *x*+1/2, −*y*+3/2, *z*−1/2; (ii) −*x*+3/2, *y*−1/2, −*z*+1/2; (iii) *x*+1, *y*, *z*; (iv) *x*+1/2, −*y*+3/2, *z*+1/2; (v) *x*−1/2, −*y*+3/2, *z*−1/2.

### Bis[diaquafluorido(1,10-phenanthroline-κ2N,N')copper(II)] hexafluoridohafnate(IV) dihydrate (IV) Crystal data

**Table:**

[CuF(C_12_H_8_N_2_)(H_2_O)_2_]_2_[HfF_6_]·2H_2_O	*F*(000) = 900
*M**_r_* = 926.07	*D*_x_ = 2.041 Mg m^−^^3^
Monoclinic, *P*2_1_/*n*	Mo *K*α radiation, λ = 0.71073 Å
*a* = 13.6451 (2) Å	Cell parameters from 9864 reflections
*b* = 7.1161 (1) Å	θ = 3.2–31.6°
*c* = 15.7457 (3) Å	µ = 4.93 mm^−^^1^
β = 99.691 (1)°	*T* = 100 K
*V* = 1507.09 (4) Å^3^	Block, blue
*Z* = 2	0.16 × 0.16 × 0.10 mm

### Bis[diaquafluorido(1,10-phenanthroline-κ2N,N')copper(II)] hexafluoridohafnate(IV) dihydrate (IV) Data collection

**Table:**

Bruker Kappa APEX CCD area detector diffractometer	5034 independent reflections
Radiation source: sealed tube	4982 reflections with *I* > 2σ(*I*)
Triumph monochromator	*R*_int_ = 0.033
Detector resolution: 8 pixels mm^-1^	θ_max_ = 31.6°, θ_min_ = 1.8°
ω and φ scans	*h* = −20→20
Absorption correction: multi-scan (SADABS; Bruker, 2016)	*k* = −10→10
*T*_min_ = 0.489, *T*_max_ = 0.746	*l* = −23→23
123138 measured reflections	

### Bis[diaquafluorido(1,10-phenanthroline-κ2N,N')copper(II)] hexafluoridohafnate(IV) dihydrate (IV) Refinement

**Table:**

Refinement on *F*^2^	Hydrogen site location: mixed
Least-squares matrix: full	H atoms treated by a mixture of independent and constrained refinement
*R*[*F*^2^ > 2σ(*F*^2^)] = 0.014	*w* = 1/[σ^2^(*F*_o_^2^) + (0.0117*P*)^2^ + 1.360*P*] where *P* = (*F*_o_^2^ + 2*F*_c_^2^)/3
*wR*(*F*^2^) = 0.035	(Δ/σ)_max_ = 0.002
*S* = 1.15	Δρ_max_ = 0.67 e Å^−^^3^
5034 reflections	Δρ_min_ = −0.70 e Å^−^^3^
230 parameters	Extinction correction: SHELXL2018/3 (Sheldrick 2015b), Fc^*^=kFc[1+0.001xFc^2^λ^3^/sin(2θ)]^-1/4^
0 restraints	Extinction coefficient: 0.00177 (14)
Primary atom site location: dual	

### Bis[diaquafluorido(1,10-phenanthroline-κ2N,N')copper(II)] hexafluoridohafnate(IV) dihydrate (IV) Special details

**Table:**

Geometry. All esds (except the esd in the dihedral angle between two l.s. planes) are estimated using the full covariance matrix. The cell esds are taken into account individually in the estimation of esds in distances, angles and torsion angles; correlations between esds in cell parameters are only used when they are defined by crystal symmetry. An approximate (isotropic) treatment of cell esds is used for estimating esds involving l.s. planes.

### Bis[diaquafluorido(1,10-phenanthroline-κ2N,N')copper(II)] hexafluoridohafnate(IV) dihydrate (IV) Fractional atomic coordinates and isotropic or equivalent isotropic displacement parameters (Å^2^)

**Table:**

	*x*	*y*	*z*	*U*_iso_*/*U*_eq_	
Cu1	0.62127 (2)	0.64187 (2)	0.68925 (2)	0.01005 (3)	
F1	0.76115 (6)	0.65941 (11)	0.70156 (5)	0.01498 (14)	
O1	0.62607 (8)	0.45374 (15)	0.78219 (7)	0.01690 (18)	
H1A	0.624 (2)	0.491 (3)	0.8306 (18)	0.038 (7)*	
H1B	0.6623 (18)	0.375 (4)	0.7848 (15)	0.031 (6)*	
O2	0.61132 (8)	0.88111 (16)	0.76902 (8)	0.0210 (2)	
H2A	0.5660 (19)	0.907 (4)	0.7866 (16)	0.035 (6)*	
H2B	0.6525 (18)	0.961 (3)	0.7795 (15)	0.028 (6)*	
N1	0.47279 (8)	0.62122 (15)	0.66010 (7)	0.01255 (18)	
N2	0.60647 (8)	0.72932 (15)	0.56647 (7)	0.01210 (18)	
C1	0.40802 (10)	0.5731 (2)	0.71056 (9)	0.0173 (2)	
H1	0.431969	0.536685	0.768377	0.021*	
C2	0.30508 (11)	0.5746 (2)	0.68062 (10)	0.0229 (3)	
H2	0.260252	0.542670	0.718419	0.027*	
C3	0.26932 (10)	0.6223 (2)	0.59655 (11)	0.0227 (3)	
H3	0.199828	0.621543	0.575619	0.027*	
C4	0.33664 (10)	0.67219 (19)	0.54175 (9)	0.0169 (2)	
C5	0.43786 (9)	0.67246 (17)	0.57741 (8)	0.0125 (2)	
C6	0.30786 (11)	0.7249 (2)	0.45299 (10)	0.0218 (3)	
H6	0.239621	0.721580	0.427555	0.026*	
C7	0.37640 (12)	0.7791 (2)	0.40497 (9)	0.0212 (3)	
H7	0.355271	0.814434	0.346577	0.025*	
C8	0.48019 (11)	0.78432 (18)	0.44052 (8)	0.0161 (2)	
C9	0.51006 (9)	0.72954 (17)	0.52666 (8)	0.0122 (2)	
C10	0.55590 (12)	0.84042 (19)	0.39478 (9)	0.0204 (3)	
H10	0.539638	0.879121	0.336361	0.024*	
C11	0.65340 (12)	0.8386 (2)	0.43549 (9)	0.0206 (3)	
H11	0.704981	0.875387	0.405306	0.025*	
C12	0.67606 (10)	0.78186 (19)	0.52197 (8)	0.0162 (2)	
H12	0.743579	0.781115	0.549530	0.019*	
Hf1	0.500000	0.500000	1.000000	0.01221 (3)	
F2	0.41065 (9)	0.67878 (17)	0.92652 (8)	0.0399 (3)	
F3	0.54442 (8)	0.69971 (16)	1.08602 (7)	0.0347 (3)	
F4	0.61050 (7)	0.56285 (16)	0.93645 (6)	0.0286 (2)	
O3	0.44170 (9)	0.97485 (17)	0.82435 (8)	0.0211 (2)	
H3A	0.4338 (19)	0.893 (4)	0.8558 (17)	0.044 (7)*	
H3B	0.4502 (18)	1.063 (4)	0.8509 (16)	0.034 (6)*	

### Bis[diaquafluorido(1,10-phenanthroline-κ2N,N')copper(II)] hexafluoridohafnate(IV) dihydrate (IV) Atomic displacement parameters (Å^2^)

**Table:**

	*U*^11^	*U*^22^	*U*^33^	*U*^12^	*U*^13^	*U*^23^
Cu1	0.00921 (6)	0.01226 (7)	0.00874 (6)	0.00025 (5)	0.00170 (5)	0.00021 (5)
F1	0.0105 (3)	0.0159 (4)	0.0182 (4)	0.0005 (3)	0.0014 (3)	−0.0001 (3)
O1	0.0223 (5)	0.0169 (4)	0.0126 (4)	0.0065 (4)	0.0061 (4)	0.0035 (3)
O2	0.0179 (5)	0.0181 (5)	0.0301 (5)	−0.0050 (4)	0.0127 (4)	−0.0111 (4)
N1	0.0116 (4)	0.0135 (5)	0.0128 (4)	−0.0001 (4)	0.0027 (3)	−0.0023 (4)
N2	0.0146 (4)	0.0112 (4)	0.0107 (4)	−0.0009 (4)	0.0028 (3)	−0.0007 (3)
C1	0.0155 (5)	0.0213 (6)	0.0166 (6)	−0.0021 (5)	0.0070 (4)	−0.0041 (5)
C2	0.0147 (6)	0.0280 (7)	0.0281 (7)	−0.0020 (5)	0.0099 (5)	−0.0065 (6)
C3	0.0117 (5)	0.0253 (7)	0.0305 (7)	0.0023 (5)	0.0024 (5)	−0.0074 (6)
C4	0.0136 (5)	0.0146 (5)	0.0210 (6)	0.0034 (4)	−0.0014 (4)	−0.0046 (5)
C5	0.0127 (5)	0.0107 (5)	0.0134 (5)	0.0020 (4)	0.0001 (4)	−0.0026 (4)
C6	0.0203 (6)	0.0179 (6)	0.0230 (6)	0.0072 (5)	−0.0084 (5)	−0.0047 (5)
C7	0.0291 (7)	0.0151 (6)	0.0157 (6)	0.0074 (5)	−0.0075 (5)	−0.0020 (5)
C8	0.0256 (6)	0.0104 (5)	0.0108 (5)	0.0033 (5)	−0.0008 (4)	−0.0006 (4)
C9	0.0161 (5)	0.0089 (5)	0.0109 (5)	0.0014 (4)	0.0004 (4)	−0.0012 (4)
C10	0.0371 (8)	0.0129 (5)	0.0112 (5)	0.0010 (5)	0.0044 (5)	0.0012 (4)
C11	0.0323 (7)	0.0161 (6)	0.0156 (6)	−0.0021 (5)	0.0104 (5)	0.0006 (5)
C12	0.0206 (6)	0.0149 (5)	0.0144 (5)	−0.0029 (5)	0.0064 (4)	−0.0013 (4)
Hf1	0.01420 (4)	0.01322 (4)	0.01094 (4)	−0.00344 (2)	0.00713 (2)	−0.00266 (2)
F2	0.0325 (6)	0.0375 (6)	0.0482 (7)	0.0045 (5)	0.0029 (5)	0.0185 (5)
F3	0.0321 (5)	0.0372 (6)	0.0387 (6)	−0.0155 (4)	0.0174 (4)	−0.0263 (5)
F4	0.0268 (5)	0.0418 (6)	0.0213 (4)	−0.0156 (4)	0.0165 (4)	−0.0086 (4)
O3	0.0212 (5)	0.0188 (5)	0.0250 (5)	−0.0002 (4)	0.0093 (4)	−0.0061 (4)

### Bis[diaquafluorido(1,10-phenanthroline-κ2N,N')copper(II)] hexafluoridohafnate(IV) dihydrate (IV) Geometric parameters (Å, º)

**Table:**

Cu1—F1	1.8899 (8)	C5—C9	1.4279 (18)
Cu1—O1	1.9763 (10)	C6—H6	0.9500
Cu1—O2	2.1335 (11)	C6—C7	1.354 (2)
Cu1—N1	2.0060 (11)	C7—H7	0.9500
Cu1—N2	2.0085 (11)	C7—C8	1.433 (2)
O1—H1A	0.81 (3)	C8—C9	1.4042 (17)
O1—H1B	0.74 (3)	C8—C10	1.413 (2)
O2—H2A	0.74 (3)	C10—H10	0.9500
O2—H2B	0.80 (3)	C10—C11	1.376 (2)
N1—C1	1.3290 (16)	C11—H11	0.9500
N1—C5	1.3588 (16)	C11—C12	1.4039 (19)
N2—C9	1.3586 (16)	C12—H12	0.9500
N2—C12	1.3254 (16)	Hf1—F2	1.9922 (11)
C1—H1	0.9500	Hf1—F2^i^	1.9921 (11)
C1—C2	1.4044 (19)	Hf1—F3	1.9863 (10)
C2—H2	0.9500	Hf1—F3^i^	1.9863 (10)
C2—C3	1.374 (2)	Hf1—F4	1.9957 (9)
C3—H3	0.9500	Hf1—F4^i^	1.9957 (9)
C3—C4	1.408 (2)	O3—H3A	0.78 (3)
C4—C5	1.4006 (17)	O3—H3B	0.75 (3)
C4—C6	1.436 (2)		
			
F1—Cu1—O1	93.54 (4)	C4—C6—H6	119.5
F1—Cu1—O2	92.88 (4)	C7—C6—C4	121.08 (13)
F1—Cu1—N1	172.75 (4)	C7—C6—H6	119.5
F1—Cu1—N2	90.83 (4)	C6—C7—H7	119.4
O1—Cu1—O2	95.86 (5)	C6—C7—C8	121.29 (13)
O1—Cu1—N1	91.49 (5)	C8—C7—H7	119.4
O1—Cu1—N2	155.24 (5)	C9—C8—C7	118.50 (13)
N1—Cu1—O2	91.79 (4)	C9—C8—C10	116.91 (13)
N1—Cu1—N2	82.44 (4)	C10—C8—C7	124.59 (13)
N2—Cu1—O2	108.26 (5)	N2—C9—C5	116.56 (11)
Cu1—O1—H1A	118.1 (17)	N2—C9—C8	123.16 (12)
Cu1—O1—H1B	119.3 (18)	C8—C9—C5	120.27 (12)
H1A—O1—H1B	109 (2)	C8—C10—H10	120.2
Cu1—O2—H2A	124 (2)	C11—C10—C8	119.50 (12)
Cu1—O2—H2B	125.3 (17)	C11—C10—H10	120.2
H2A—O2—H2B	110 (3)	C10—C11—H11	120.3
C1—N1—Cu1	129.04 (9)	C10—C11—C12	119.49 (13)
C1—N1—C5	118.73 (11)	C12—C11—H11	120.3
C5—N1—Cu1	112.17 (8)	N2—C12—C11	122.24 (13)
C9—N2—Cu1	112.16 (8)	N2—C12—H12	118.9
C12—N2—Cu1	129.15 (9)	C11—C12—H12	118.9
C12—N2—C9	118.69 (11)	F2^i^—Hf1—F2	180.0
N1—C1—H1	119.1	F2—Hf1—F4	90.34 (5)
N1—C1—C2	121.71 (13)	F2—Hf1—F4^i^	89.66 (5)
C2—C1—H1	119.1	F2^i^—Hf1—F4^i^	90.34 (5)
C1—C2—H2	120.1	F2^i^—Hf1—F4	89.66 (5)
C3—C2—C1	119.88 (13)	F3^i^—Hf1—F2^i^	91.48 (6)
C3—C2—H2	120.1	F3^i^—Hf1—F2	88.52 (6)
C2—C3—H3	120.3	F3—Hf1—F2^i^	88.52 (6)
C2—C3—C4	119.36 (13)	F3—Hf1—F2	91.48 (6)
C4—C3—H3	120.3	F3^i^—Hf1—F3	180.0
C3—C4—C6	124.23 (13)	F3—Hf1—F4	90.69 (4)
C5—C4—C3	117.12 (13)	F3^i^—Hf1—F4^i^	90.69 (4)
C5—C4—C6	118.64 (13)	F3^i^—Hf1—F4	89.31 (4)
N1—C5—C4	123.15 (12)	F3—Hf1—F4^i^	89.31 (4)
N1—C5—C9	116.67 (11)	F4^i^—Hf1—F4	180.0
C4—C5—C9	120.18 (12)	H3A—O3—H3B	107 (3)

Symmetry code: (i) −*x*+1, −*y*+1, −*z*+2.

### Bis[diaquafluorido(1,10-phenanthroline-κ2N,N')copper(II)] hexafluoridohafnate(IV) dihydrate (IV) Hydrogen-bond geometry (Å, º)

**Table:**

*D*—H···*A*	*D*—H	H···*A*	*D*···*A*	*D*—H···*A*
O1—H1*A*···F4	0.81 (3)	1.78 (3)	2.5926 (14)	176 (3)
O1—H1*B*···F1^ii^	0.74 (3)	1.85 (3)	2.5861 (13)	172 (3)
O2—H2*A*···O3	0.74 (3)	1.95 (3)	2.6906 (15)	176 (3)
O2—H2*B*···F1^iii^	0.80 (3)	1.83 (3)	2.6255 (13)	175 (2)
O3—H3*A*···F2	0.78 (3)	1.94 (3)	2.7270 (17)	176 (3)
O3—H3*B*···F3^iv^	0.75 (3)	1.96 (3)	2.7020 (15)	173 (3)

Symmetry codes: (ii) −*x*+3/2, *y*−1/2, −*z*+3/2; (iii) −*x*+3/2, *y*+1/2, −*z*+3/2; (iv) −*x*+1, −*y*+2, −*z*+2.
